# Challenges and opportunities in next-generation LED therapeutic devices

**DOI:** 10.1038/s41377-025-01990-z

**Published:** 2025-09-15

**Authors:** Chenxi Wang, Qiang Yu, Ming Li, Haoyi Chen, Huizhen Fan, Yingying Ma, Zhitao Zhang, Mei X. Wu, Min Lu

**Affiliations:** 1 Department of Orthopaedics, Shanghai Key Laboratory for Prevention and Treatment of Bone and Joint Diseases, Shanghai Institute of Traumatology and Orthopaedics, Ruijin Hospital, Shanghai Jiao Tong University School of Medicine, Shanghai, China; 2 State Key Laboratory of Synergistic Chem-Bio Synthesis, School of Chemistry and Chemical Engineering, Zhangjiang Institute for Advanced Study, Shanghai Jiao Tong University, Shanghai, China; 3 Wellman Center for Photomedicine, Massachusetts General Brigham, Harvard Medical School, Havard University, Cambridge, MA USA

**Keywords:** Organic LEDs, Inorganic LEDs

## Abstract

Phototherapy offers advantages of non-invasiveness, cost-effectiveness, localized treatment, and potential for home-based care across various medical conditions. However, its adoption is hindered by the large size, limited safety, and professional operation requirements of current phototherapeutic devices. Unlike bulky laser phototherapeutic devices, wearable and implantable LED-based devices overcome these limitations, offering improved safety, portability, and uniform light distribution, making them promising prototypes for next-generation phototherapies. This review explores the home-care potentials of phototherapy from a clinical application perspective and provides a comprehensive overview of its therapeutic mechanisms and diverse applications. By synthesizing the latest advancements and cutting-edge research, we identify key clinical challenges associated with wearable and implantable phototherapy devices and propose fundamental strategies to address these limitations, such as miniaturization, biocompatibility, and energy efficiency. Furthermore, we draw on interdisciplinary cutting-edge research to address the challenges faced by phototherapy devices. We also emphasize the critical value of integrating artificial intelligence (AI) and flexible sensing technologies within phototherapy systems. Specific methods and potential applications are discussed for effectively integrating phototherapy systems with AI algorithms to establish a closed-loop diagnostic and therapeutic system. Grounded in clinical applications, we outline concrete research directions for developing next-generation LED-based phototherapy devices. This review delivers valuable insights for clinicians leveraging phototherapy and offers a roadmap for researchers in material science, flexible electronics, and AI, fostering interdisciplinary innovations to advance future phototherapy applications.

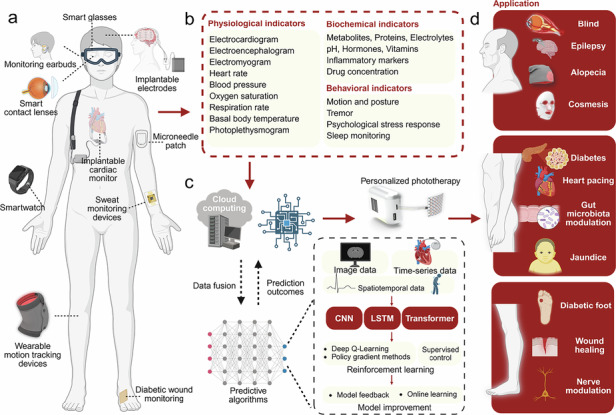

## Abstract (web summary)

This review highlights recent progress and challenges in wearable and implantable LED-based therapeutic devices, along with potential solutions. It also outlines clinical requirements and manufacturing pathways for future phototherapy systems.

## Introduction

The application of phototherapy dates back 3500 years, when ancient Egyptians and other civilizations along the Nile River combined medicinal plants with sunlight to treat vitiligo^1^. In the late 19th century, sunlight was found to have therapeutic effects on anthrax and rickets. In the early 20th century, Niels Ryberg Finsen utilized artificial light sources to treat cutaneous tuberculosis (lupus vulgaris), earning the Nobel Prize in Physiology or Medicine in 1903^2^. In 1960, Dr. Maiman developed the solid-state laser and performed the first retinal tumor surgery using laser technology^3^. Due to its high brightness, monochromaticity, and directionality, laser therapy has been widely applied in treating pigmented disorders^4^, tumors^5^, scars^6^, infections^7^, vascular malformations^4^, and ophthalmic surgeries^8^. An important development occurred in photomedicine in 1983 when Rox Anderson introduced the concept of selective photothermolysis^9^. Building on its historical foundation, phototherapy has evolved into a diverse and versatile field, leveraging various light wavelengths for specific therapeutic applications.

**Fig. 1:**
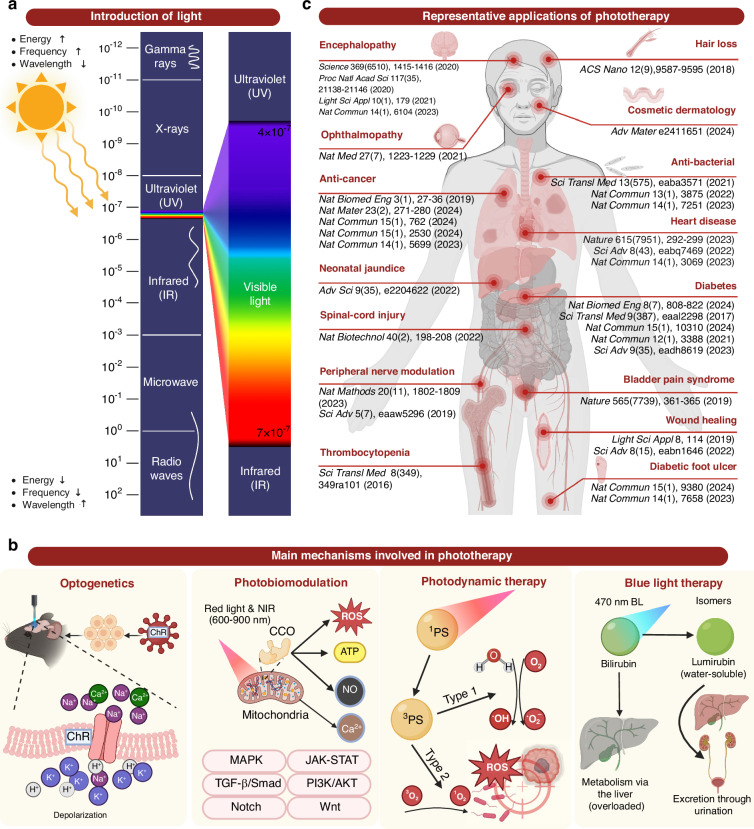
**Phototherapy and the underlying mechanisms**. **a** Introduction of light. The electromagnetic spectrum illustrates the distribution of electromagnetic waves across various wavelengths. The enlarged section corresponds to the ultraviolet, visible, and infrared regions, which are the primary wavelengths used in phototherapy applications. **b** Schematic representation of the main mechanisms involved in phototherapy, including optogenetics^24^, photobiomodulation^27^, photodynamic therapy^30^, and blue light therapy^13^. Optogenetics activates light-sensitive proteins, such as channelrhodopsin-2 (ChR2), allowing significant cation influx, particularly Na^+^, through ion channels, leading to depolarization and neuronal excitation. Photobiomodulation employs red and near infrared (NIR) light to activate cytochrome C oxidase (CCO) in the mitochondrial electron transport chain, enhancing ATP synthesis and reducing oxidative stress while upregulating signaling molecules such as nitric oxide (NO) and calcium ions (Ca^2+^). ATP, adenosine triphosphate. Photodynamic therapy uses light of specific wavelengths to activate photosensitizers (PSs), generating reactive oxygen species (•OH, •O_2_^-^, and ^1^O_2_) to eliminate cancers and pathogens. Blue light phototherapy enhances bilirubin absorption in the skin, converting it into water-soluble lumirubin, which is excreted from the body without hepatic metabolism. **c** Representative applications of diseases treated with phototherapy and their corresponding research references: encephalopathy^25,49–51^, ophthalmopathy^37^, anti-cancer^58,150,165–167^, neonatal jaundice^13^, spinal-cord injury^52^, peripheral nerve modulation^53,54^, thrombocytopenia^168^, hair loss^18^, cosmetic dermatology^31^, anti-bacterial^7,169,170^, heart disease^19,41,42^, diabetes^43–47^, bladder pain syndrome^48^, wound healing^17,34^, and diabetic foot ulcer^35,36^. Figure 1, created with BioRender.com, released under a Creative Commons Attribution-NonCommercial-NoDerivs 4.0 International license

Each segment of the electromagnetic spectrum offers distinct biological effects and clinical advantages based on its unique energy, frequency, and wavelength (Fig. 1a). In the gamma-ray range ( < 0.01 nm), high-energy radiation targets tumors in radiotherapy^10^, while X-rays (0.01 ~ 10 nm) are used for diagnostic imaging^11^. Ultraviolet (UV) light (10 ~ 400 nm) addresses skin conditions and tumors^12^. Blue light (450 ~ 490 nm) is used for antibacterial therapy^7^ and neonatal jaundice treatment^13^, while green light (495 ~ 570 nm) aids in retinal therapy^14^ and pain relief^15^. Yellow light (570 ~ 590 nm) enhances immune function and improves mood^16^, and red light (620 ~ 750 nm) promotes wound healing^17^, hair growth^18^, and deep-tissue phototherapy^19^. Near-infrared lights (NIR, 700 ~ 2500 nm), particularly 808 and 980 nm, are effective for pain management, and technologies like functional near-infrared spectroscopy are used for tissue imaging^20^. Mid-infrared lights (2500 ~ 25,000 nm) include CO₂ lasers (10,600 nm) for surgical procedures^21^, while far-infrared lights ( > 25,000 nm) generate thermal effects for conditions like arthritis^22^ **(**Fig. 1a).

The phototherapeutic mechanisms primarily rely on several pathways (Fig. 1b). Optogenetics^23^ is a technique that allows precise control of neural activity and cellular functions through the activation of specific light-sensitive proteins, including ChR2, enhanced bacteriorhodopsin, natronomonas pharaonis halorhodopsin, and the like^24^. When exposed to a 473 nm blue light, ChR2 channels open, allowing a significant influx of cations like Na^+^, leading to depolarization and subsequent neuronal excitation^25^. This mechanism enables precise regulation of neuronal and cellular functions, making optogenetics a powerful tool widely used in neuroscience and biomedical research (Fig. 1b). Photobiomodulation (PBM)^26^, previously referred to as low-level laser therapy (LLLT), typically employs red and NIR (600 ~ 980 nm) to activate the rate-limiting enzyme cytochrome C oxidase (CCO) in the mitochondrial electron transport chain. This activation enhances ATP synthesis and reduces oxidative stress while upregulating signaling molecules such as nitric oxide (NO) and calcium ions (Ca^2+^). Consequently, a cascade of downstream signaling pathways is triggered, resulting in the regulation of cellular physiological functions^27^ **(**Fig. 1b**)**. Photodynamic therapy (PDT)^28,29^ uses light of a specific wavelength to activate photosensitizers (PSs), generating reactive oxygen species (•OH, •O_2_^-^, and ^1^O_2_) to eliminate cancers and pathogens^30^. Blue light phototherapy, utilizing the wavelength of 400 ~ 470 nm, facilitates the absorption of light energy by bilirubin molecules in the skin and converts it into water-soluble isomers, such as lumirubin. These isomers can be excreted from the body without requiring hepatic metabolism^13^ (Fig. 1b). These mechanisms underpin the therapeutic efficacy of phototherapy across diverse medical scenarios.

Current phototherapy has been successfully applied to several major human organs, including skin, nervous, circulatory, urinary, musculoskeletal, and digestive systems **(**Fig. 1c, Table 1). Due to the limited penetration of light, phototherapy is primarily focused on the body surface, such as hair loss^18^, dermatological diseases^31–33^, wound healing^17,34^, chronic wound management^35,36^, ophthalmopathy^37^, and neonatal jaundice^13,38^ (Fig. 1c, Table 1). Such devices are designed to fit the body surface (e.g., skin, mucous membranes) and deliver light energy to superficial tissues, as well as areas that can be penetrated by external light sources, such as subcutaneous capillaries and the cerebral cortex. The therapeutic targets are typically located in the epidermis, dermis, shallow subcutaneous layers, or areas that can be penetrated by near-infrared light, typically up to 3–5 cm beneath the skin^39^. Since they deliver light to the target area without requiring surgical intervention, these devices are clinically defined as wearable phototherapy devices. For instance, Sahel et al.^37^ injected an adeno-associated viral vector encoding ChrimsonR into the eye, to facilitate partial vision restoration in a blind patient through the combined use of optogenetics and phototherapy goggles (Fig. 1c). Additionally, the face-fit surface-lighting micro light-emitting diodes (micro-LED) mask developed by Kim et al.^31^ conforms to complex facial contours, giving rise to significant improvement in facial elasticity, sagging, and wrinkles (Table 1). Analogously, the textile-based blue organic light-emitting diodes (OLEDs) developed by Choi et al.^13^ balance comfort and therapeutic efficacy, enabling at-home treatment for neonatal jaundice (Fig. 1c). Recently, we fabricated a stretchable red and blue LED (r&bLED) patch^36^, which potentially offers a convenient antibacterial and wound-healing facilitation for managing chronic infectious wounds at home (Fig. 1c). Furthermore, a randomized controlled trial validated the efficacy of LLLT in the treatment of traumatic brain injury. Among 68 randomly assigned patients, 33 patients received NIR light therapy using a custom LED-helmet within 72 h post-injury, while 35 patients received sham treatment as controls. The results demonstrated statistically significant changes in multiple brain diffusion tensor imaging parameters during the subacute phase^39^.

**Table 1:** Summary of Recent Advances in Flexible Phototherapy Devices

Phototherapy mechanism	Clinical application	Light emitting material	Light parameters		Substrate	Device type	Highlights	Refs
			Wavelength	Power				
Optogenetics	Nerve stimulation	OLED	400 ~ 580 nm	0.5 mW mm^-2^	Parylene C	Implantable	Ultra-flexible film OLEDs provide precise light stimulation to the brain and can conform to various anatomical structures.	^25^
	Vision loss	OLED	600 nm	0.1 mW mm^-2^	Silicon	Wearable	High-brightness, highly directional OLEDs have been incorporated into wearable prosthetics to facilitate optogenetic therapy for retinal cells.	^156^
	Control of cardiac rhythms	Micro-LED	591 nm	0.1 mW mm^-2^		Wearable	The 591 nm micro-LED was integrated into a wearable textile vest, enabling non-invasive optogenetic control of heart rate in mice through the chest wall.	^41^
	Diabetes	LED	545 nm	0.15 mW cm^-2^		Wearable	An innovative approach combining smartwatches with green light control provides new possibilities for the precise regulation of transdermal therapeutic gene delivery.	^47^
	Bladder pain syndrome	Micro-inorganic LED	530 nm	3.3 or 10 mW mm^-2^	Silicone	Implantable	An innovative wireless closed-loop system has been developed for the optogenetic modulation of peripheral nerves.	^48^
	Cardiac pacing	Micro-LED array	Blue light	Pulse width of 10 ms, 101 mW mm^-2^	PI	Implantable	A customized soft, thin-film micro-LED array enables high spatiotemporal precision in optogenetic stimulation delivery, while a closed-loop system allows for rapid pacing or defibrillation upon detection of arrhythmias.	^42^
	Spinal cord injury	Micro-LED	470 nm 535 nm	Pulse width of 10 ms, 50 mW mm^-2^	PI	Implantable	Optogenetic stimulation is applied to the entire spinal cord region without the use of wires, while the closed-loop system enables real-time monitoring of physiological responses in mice and automatic adjustment of light stimulation based on feedback.	^52^
	Diabetes	LED	660 nm	20 mW cm^-2^		Wearable	An optogenetic switch responsive to red and far-red light has been developed, offering high controllability for precise regulation of target genes.	^44^
	Diabetes	LED	730 nm	1 mW cm^-2^		Implantable	Remote control of optogenetic cells is enabled via a smartphone, enhancing operational flexibility and improving patient autonomy in diabetes management.	^45^
PBM	Wound healing	OLED	629 nm 534 nm 466 nm	5 mW cm^-2^	Cylindrical-shaped materials, textiles and paper	Wearable	Sandwich-structured transferable OLEDs can be applied to various flexible substrates, such as textiles, enabling them to conform to the complex surfaces of human skin.	^34^
	Ischemic stroke	LED array	630 nm	17 mW cm^-2^	PI	Implantable	An implantable multi-LED array achieves stable contact with the target cortical region and the skull for PBM. Optimal results indicate that 630 nm is most effective in reducing infarct volume and neuronal damage following ischemic stroke.	^55^
	Skin anti-aging	Micro-LED	627 nm	19.78 μW cm^−2^	GaAs	Wearable	The face-fit surface-lighting µLED mask conforms to complex facial contours (elevations and curves), enhancing the effectiveness of PBM and resulting in significant improvements in facial elasticity, sagging, and wrinkles.	^31^
	Diabetic retinopathy	LED	630 ~ 1000 nm	120 µW	PET	Wearable	Intelligent wireless near-infrared emitting contact lenses can non-invasively stimulate retinal repair and regeneration, integrated with a smart control system.	^157^
	Hair-growth	Micro-LED	650 nm	∼30 mW mm^–2^	GaAs	Wearable	Monolithic flexible red vertical LEDs offer excellent light uniformity and are suitable for wearable applications aimed at stimulating hair growth.	^18^
	Melanogenesis inhibition	Micro-LED	630 nm	0.4 mW cm^−2^	Plastic substrate	Wearable	The wearable design allows users to incorporate the device more easily into their daily lives.	^62^
	Wound healing	LED array	630 nm	~13.37 mW cm^−2^	PI	Wearable	The adhesive nanofiber membrane wound dressing is combined with LED phototherapy.	^17^
	Hair-growth	OLED	640 nm	10 mW cm^−2^		Wearable	Red OLEDs have been utilized for the first time in a mouse model to validate the stimulation of hair growth and assess their effects on hair follicle cells.	^158^
	Diabetes	OLED	600 ~ 700 nm	1.33 mW cm^-2^	Parylene C	Implantable	Utilizing OLED catheters, uniform PBM within the duodenum has been achieved, with potential implications for the regulation of glucose and insulin metabolism.	^43^
		QLED	620 nm	∼8 mW cm^−2^	Glass	Wearable	The narrow emission band and wavelength tunability of QLEDs made it possible to fit the emission spectrum into the absorption window of cytochrome C (for PBM).	^113^
	Hair growth	QD-LED	630, 700 and 730 nm	>23.28 mW·cm^−2^	PET	Wearable	Wearable QD-OLED patch is developed for real-time wavelength controllable high-power NIR photomedicine. NIR QD-OLED demonstrated that it could increase the proliferation of HFDP cells by up to 131% through NIR wavelength control.	^114^
	Wound healing	QD-LED	NIR	2.5 mW cm^-2^		Wearable	A red/NIR light source that matches 81.7% of the absorption spectrum of CCO enzyme was produced. Exposure of the produced light source to the wound area accelerated wound healing.	^115^
PDT	Anticancer	OLED	Color-tunable	>100 mW cm^-2^		Wearable	The parallel stacked OLED structure enhances light output intensity and therapeutic effectiveness while also possessing color adjustment capabilities.	^159^
	Wound management & antibacterial	OLED	669 ~ 737 nm	>9 mW cm^-2^	PET	Wearable	Using flexible OLED as the light source enables uniform illumination for PDT. When combined with methylene blue as a photosensitizer, this approach effectively eliminates over 99% of *S. aureus*.	^160^
	Long-term autonomous cancer therapy	Miniature LED	470 nm		PET	Implantable	An implantable system that integrates human motion with PDT has been developed, creating a self-powered treatment solution that reduces reliance on external power sources.	^127^
	Anti-cancer	LED	630, 530 and 460 nm	<100 μW cm^-2^	PDMS	Implantable	An implantable, wirelessly powered PDT system utilizes tissue-adhesive optoelectronics that securely adhere to the internal tissue surface without the need for surgical sutures.	^58^
	Oral cancer treatment or diabetic wound repairs	QLED	620 nm		PEN	Wearable	The first in vitro study demonstrates that QLED-based photodynamic therapy effectively eradicates methicillin-resistant *Staphylococcus aureus*. High-efficiency QLED featuring narrow emission spectra and specific peak wavelengths achieves a luminance exceeding 20,000 cd m^-^² under a low driving voltage of 6 V.	^112^
Blue light therapy	Neonatal jaundice	OLED	470 nm	>20 µW cm^-2^ nm^-1^	Textile	Wearable	Textile-based blue OLEDs combine wearable technology with phototherapy, offering a convenient at-home treatment solution for neonatal jaundice.	^13^
UVA therapy	Dermatological diseases	LED	360 nm	20 ~ 80 mW cm^-2^	PI	Wearable	The integration of PLGA microneedles for light conduction enhances the depth of illumination.	^32^

To date, researchers are devoting enormous efforts to overcoming the challenges of delivering light to deeper tissues^40^, with the ultimate goal of expanding the applicability of phototherapy. Among them, fiber-optic and implantable LED devices are being developed to treat heart diseases^19,41,42^, diabetes^43–47^, bladder pain syndrome^48^, encephalopathy^25,49–51^, spinal cord injuries^52^, and neurological disorders^53,54^ (Fig. 1c, Table 1). These devices require implantation through natural body cavities (e.g., gastrointestinal tract, oral cavity, nasal cavity), minimally invasive procedures, or open surgery, to make direct contact with or be placed near deeper tissues, enabling precise light energy delivery. From a clinical application perspective, these devices are commonly referred to as implantable phototherapy devices. For example, Ausra et al.^42^ customized a soft, thin-film micro-LED array that enabled high spatiotemporal precision for optogenetic stimulation delivery, facilitating cardiac pacing and defibrillation when implanted in vivo (Fig. 1c). Additionally, the combination of µLED implants with optogenetically modified neurons expressing channelrhodopsin-2 (ChR2) allows for facile neural modulation within the body^25,46,48,52,53^ (Table 1). Kim et al.^55^ developed an implantable multi-LED array that ensures stable contact with the target cortical region and skull for PBM therapy (Table 1). Their studies demonstrated that 630 nm red light effectively reduced infarct volume and neuronal damage following ischemic stroke. Besides, Kathe et al.^52^ developed a µLED system capable of conforming to the dura mater of the spinal cord. Using an optogenetic model of spinal cord injury in mice, they integrated a physiological signal sensing module with a phototherapy module to achieve closed-loop control of spinal cord neurons. This innovative approach effectively addressed challenges associated with spinal-cord injuries (Fig. 1c, Table 1).

Over the past five years, the development of wearable and implantable phototherapy devices has accelerated significantly, with their effectiveness in treating various diseases being well demonstrated. Despite significant advancements, wearable and implantable phototherapy devices still face several challenges. Different diseases require specific parameters (wavelength, power density, and exposure duration) yet current devices often lack the precision and monitoring system to meet diverse clinical needs. Long-term comfort, biocompatibility, and energy supply also require optimization. Furthermore, improving therapeutic efficiency, expanding applications, and reducing costs in complex clinical settings remain pressing issues. Addressing these technical bottlenecks and aligning devices with clinical requirements is essential for advancing this field.

## Challenges and unmet clinical needs in phototherapy devices

**Fig. 2:**
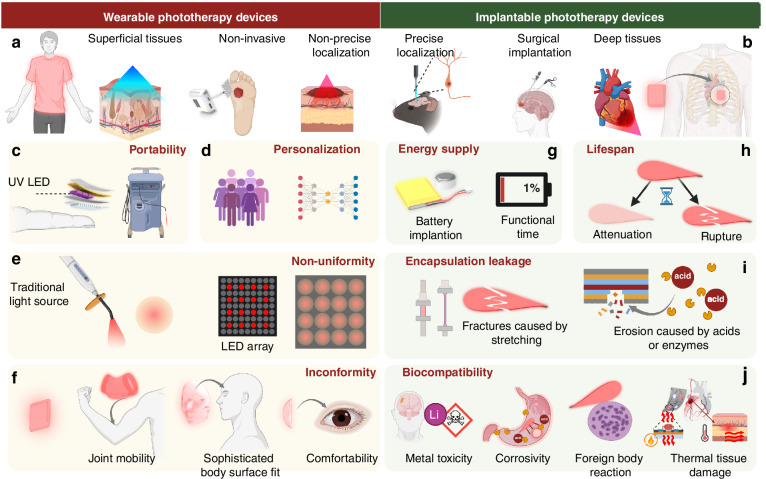
**Challenges in wearable and implantable phototherapy devices**. **a** Wearable phototherapy devices for superficial tissues, emphasizing portability, personalization, and energy supply. **b** Implantable phototherapy devices for deep tissues, focusing on precise localization, energy supply, and biocompatibility. Challenges of wearable phototherapy devices in clinical applications: **c** Lack of portability^32,33^. **d** Inadequate personalization to meet individual needs^57^. **e** Insufficient uniformity of illumination from current light source devices, including traditional light source and LED array^17,19^ (reprinted with permission^32^. Copyright 2021, John Wiley & Sons). **f** Poor conformity of wearable devices^31^, including issues with joint mobility, sophisticated body surface fit, and lack of comfortability. Challenges of implantable phototherapy devices in clinical settings: **g** Traditional batteries as power sources raise concerns about implantation and limited functional time^48,58^. **h** Device lifespan is constrained by light output attenuation and rupture^43^.**i** Encapsulation may fail due to stretching-induced fractures or erosion from acids or enzymes, leading to leakage. **j** Biocompatibility is limited by heavy metal toxicity from batteries, in vivo corrosivity, and foreign body reactions^25^. Figure 2, created with BioRender.com, released under a Creative Commons Attribution-NonCommercial-NoDerivs 4.0 International license

As previously mentioned, researchers classify LED phototherapy devices into wearable and implantable categories from the perspective of clinical applications. The choice between wearable or implantable phototherapy strategies depends on factors such as the depth of the target tissue^40^, the nature of the disease, and whether anatomical barriers^28^ (e.g., bone structures, dense fascia) need to be crossed, as well as the need for precise spatial localization^56^ (Fig. 2a, b). Wearable devices do not require surgical intervention, providing higher patient compliance; however, they face limitations in addressing the phototherapy of deeper tissues. Implantable phototherapy devices, on the other hand, help overcome the challenge of delivering light to deeper organs and can enable precise phototherapy (e.g., optogenetic control of bladder afferent nerves^48^) (Fig. 2a, b). However, both types face numerous technical and clinical challenges necessitating efforts to improve therapeutic efficacy and user experience. From the perspective of clinical phototherapy applications, wearable devices require excellent portability, personalized phototherapy capabilities, uniform light distribution, and conformity to the contours of the body surface to enhance therapeutic efficacy (left, Fig. 2). In addition to these requirements, implantable phototherapy devices must meet challenges such as energy supply, device longevity, encapsulation leakage, biocompatibility, and so on (right, Fig. 2).

### Challenges in wearable phototherapy devices

First, insufficient portability of phototherapeutic devices significantly limits their usage scenarios (Fig. 2c). Phototherapy devices for skin diseases are often large and cumbersome, making them unsuitable for home use by patients^33^. In comparison, Zhang et al.^32^ developed a compact phototherapy device designed for skin treatment. Its enhanced portability enables patients to undergo treatment conveniently at any time, thereby improving therapeutic outcomes. Secondly, current phototherapy devices lack personalized and precise treatment capabilities (Fig. 2d). For instance, managing symptoms in epilepsy patients often relies on preventive medication or optogenetic-based phototherapy^57^. If a system could accurately predict epileptic episodes based on the patient’s physiological data and changes in brainwave patterns, timely interventions could significantly improve outcomes. Addressing this challenge is essential for future wearable phototherapy devices to enhance therapeutic efficacy and improve patient compliance. Thirdly, the uniformity of light exposure in phototherapy devices is a critical factor influencing therapeutic outcomes (Fig. 2e). Laser-based point light sources often lead to uneven light intensity, resulting in variable efficacy between central and peripheral regions^19^. Researchers have explored LED array configurations to enhance light uniformity; however, insufficient illumination persists in the gaps between LEDs^17^. Moreover, irregular surface geometries in the target illumination area further compromise the effectiveness of phototherapy (Fig. 2f). For instance, the high mobility of human joints makes conventional rigid phototherapy devices incompatible with joint movements, preventing effective therapy during motion. Similarly, the complex anatomical structures of the face and periorbital regions, coupled with significant inter-individual variability, pose challenges to achieving uniform light distribution^31^. Apparently, improving phototherapy devices to achieve better illumination uniformity, enhanced portability, and greater adaptability to body surface contours is a critical challenge that must be addressed to enhance therapeutic efficacy (Fig. 2c–f).

### Challenges in implantable phototherapy devices

Due to the limited penetration depth of light, its delivery directly to internal tissues remains formidable challenges for wearable devices^52^. On the other hand, implanted phototherapy systems pose unique energy supply challenges, as their batteries not only face implantation difficulties but also present potential biosafety concerns **(**Fig. 2g**)**. Key considerations include battery size, energy density, and biocompatibility. Mickle et al.^48^ demonstrated wireless power transfer for optogenetic therapy; however, the required coils were bulky and could only power a limited number of LEDs. In contrast, Yamagishi et al.^58^ employed a self-powered approach for high-power PDT, but challenges remain in effectively covering large treatment areas. Apparently, engineering a biocompatible, safe, and optimized energy supply tailored to the specific requirements of implantable phototherapy systems is crucial for advancing their functionality and therapeutic potential. Moreover, implantable phototherapy devices must contend with the harsh physicochemical environment. Sim et al.^43^ demonstrated the use of OLED-based phototherapy devices implanted in the small intestine for diabetes treatment. However, the vulnerability of OLED materials to water and oxygen degradation severely limits the duration of effective phototherapy (Fig. 2h). To address this issue, dense and biocompatible encapsulation techniques are required to ensure device longevity. Nevertheless, overly thick encapsulation materials can compromise light intensity and device flexibility, while excessively thin encapsulation increases the risk of harmful material leakage, raising safety concerns (Fig. 2i). Enhancing the durability of light-emitting materials and improving encapsulation quality are critical challenges for advancing implantable phototherapy devices. Most importantly, as implantable devices, phototherapy systems must ensure robust biocompatibility and safety (Fig. 2j). Materials used in batteries, light-emitting components, encapsulation layers, and conductive elements may contain metals or corrosive materials that can trigger metal toxicity, corrosive reactions, or immune-mediated foreign body responses. Kim et al.^25^ addressed these challenges by developing a flexible OLED-based phototherapy device using a soft substrate and xylene film encapsulation. This approach minimized mechanical damage to neural tissues while effectively reducing the risk of immune rejection associated with implanted devices. Besides the biocompatibility of materials, heat generated during phototherapy and by the electronic components may cause thermal damage^59^. Therefore, effective heat dissipation design is also crucial (Fig. 2j).

From the perspective of clinical phototherapy applications, future LED phototherapy devices must achieve comprehensive advancements to address the aforementioned challenges. This will require the development of novel conductive materials, light-emitting materials, encapsulation materials, and batteries, as well as advancements in manufacturing processes. Addressing these issues represents a complex interdisciplinary endeavor. In the following sections, we will propose specific strategies and highlight cutting-edge research aimed at overcoming these challenges. Additionally, we will summarize the design principles and fabrication workflows for next-generation LED phototherapy devices.

## Strategies to overcome challenges in phototherapeutic prototypes

As phototherapy technologies continue to advance in the medical field, innovations in material science and fabrication technologies have established a robust foundation for addressing technical challenges and achieving substantial improvements in device performance. Incorporating the latest advancements and clinical needs identified by phototherapy specialists, our research team has reviewed and proposed targeted strategies to tackle the current limitations of phototherapy devices. These strategies focus on enhancing light distribution uniformity, advancing device miniaturization, improving implantability, integrating diagnostic and therapeutic functionalities, and enabling the intelligent modulation of phototherapy parameters.

### Strategies to improve illumination uniformity

**Fig. 3:**
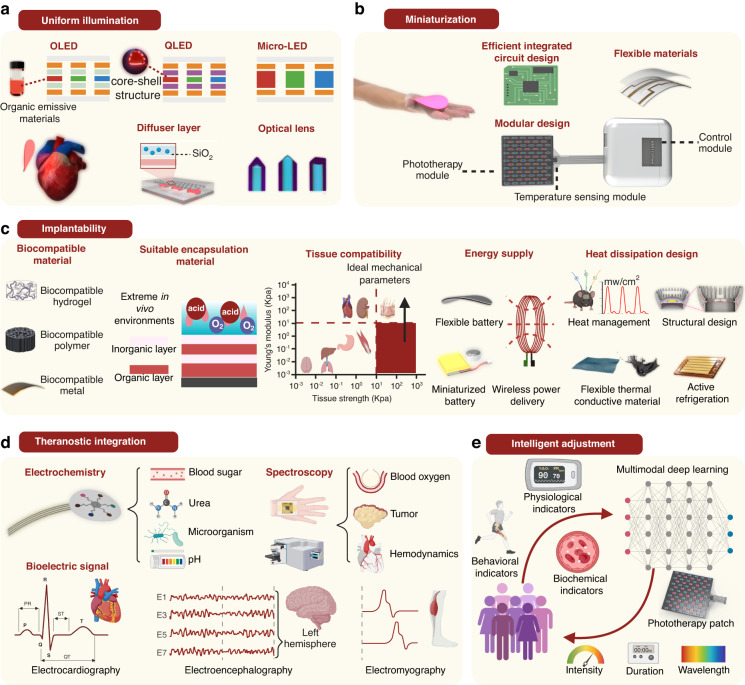
**Advanced strategies to address challenges in clinical applications of phototherapy**. **a** Enhance the uniform illumination of phototherapy *via* OLED^60^, micro-LED^31^, diffuser layer^62^, and optical lens^19^ technologies. **b** Achieving miniaturization of the overall device through effective integrated circuits^52^, flexible materials^34^, and modular design. **c** By selecting biocompatible materials, employing appropriate encapsulation strategies, ensuring the mechanical compatibility of flexible phototherapy devices with tissues, and optimizing energy supply solutions, the implantability of phototherapy devices can be enhanced (the image for structural design is reprinted with permission^75^. Copyright 2023, Nonferrous Metals Society of China. The image for active refrigeration is reprinted with permission^76^ under Creative Common CC BY license. Copyright 2022, John Wiley & Sons). **d** Realizing theranostic integration by monitoring relevant electrochemical^79^, spectroscopic^67,140,163^, and bioelectric signals^80^. **e** Employing AI to intelligently integrate detection information and output suitable phototherapy parameters^57^. Figure 3, created with BioRender.com, released under a Creative Commons Attribution-NonCommercial-NoDerivs 4.0 International license

Zhang et al.^60^ demonstrated the use of OLED technology to achieve surface light emission, resulting in improved illumination uniformity and enhanced phototherapy efficacy. QLED materials, which are compatible with the same fabrication processes as OLEDs, were utilized by Bian et al.^61^, who employed spin-coating and thermal evaporation deposition techniques to produce QLED devices capable of uniformly emitting high-intensity green light. Similarly, Kim et al.^31^ utilized µLED technology, achieving comparable uniformity in light distribution with notable improvements in therapeutic outcomes. Lee et al.^62^ introduced a diffusion layer, such as SiO₂, and Deng et al.^19^ employed fiber optic lenses, enabling phototherapy devices with initially uneven illumination to achieve significantly more uniform light distribution (Fig. 3a). This design significantly reduces the risk of localized burns caused by uneven illumination while enhancing the effectiveness of treatment for large lesions, particularly in anatomically complex areas such as the face^62^, joints^63^ and curved body surfaces^36,64^.

### Strategies for miniaturization of phototherapy devices

Miniaturization technologies are key to realizing wearable and implantable phototherapy devices (Fig. 3b). Through efficient integrated circuit designs and modular structures, the size and weight of devices have been substantially reduced. In this regard, Li et al.^36^ designed a phototherapy patch comprising a phototherapy module, sensing module, power supply module, and Bluetooth/Wi-Fi module, significantly enhancing the portability and usability (Fig. 3b). The use of soft materials further enables the device to closely conform to the human body, ensuring effective illumination in complex curved areas such as joints, thereby enhancing both comfort and therapeutic results^65^. Additionally, soft materials allow for further miniaturization of the device^66^, making it more suitable for wearable or implantable phototherapy applications.

### Strategies to enhance implantability of phototherapy devices

For implantability^55^, optimizing material selection and encapsulation technologies are crucial to ensuring both performance and biosafety. Additionally, the mechanical properties of the implantable phototherapy device and their compatibility with the target organ^41,67^, along with the choice of energy supply method^41^, are equally crucial to the design of implantable phototherapy devices (Fig. 3c). The use of biocompatible materials, such as hydrogels, flexible polymers, and biocompatible metals, significantly reduces the risks of inflammation and immune rejection^68^. Multilayer encapsulation technologies (including both inorganic and organic layers) preserve device stability under extreme in vivo conditions, such as high humidity, acidity, and oxygen levels, while preventing leakage of harmful substances^69^. Furthermore, by adjusting the Young’s modulus and tensile strength of device materials to align with the mechanical properties of human tissues, potential biosafety issues post-implantation can be minimized. Ideally, the device’s overall Young’s modulus should be lower than that of the target tissue, while its tensile strength should exceed that of the tissue^70^. Additionally, depending on the device’s expected in vivo residence time, solutions such as soft batteries^70^, micro-batteries^71^, wireless power^48^, or energy harvesting methods^58^ can be employed to address energy supply challenges **(**Fig. 3c**)**. Thermal management is critical for implantable phototherapy devices. For low level light power therapies, such as PBM^43^ or optogenetics^52^, passive heat transfer *via* body tissues suffices to maintain safe temperatures. However, high level light power applications like PDT require effective thermal management. Pulsed light therapy^72,73^ can control heat generation while maintaining efficacy. Flexible thermal conductive materials^74^, optimized heat dissipation structures^75,76^, and micro cooling plates^77^ enable passive and active cooling, ensuring the device stays within safe temperature ranges, thus enhancing performance and biosafety (Fig. 3c).

### Strategies for integrating monitoring, AI and phototherapy

The integration of therapeutic and diagnostic capabilities represents a critical advancement in phototherapy devices (Fig. 3d). By incorporating electrochemical sensors^78^ and spectroscopic analysis modules^79^, these devices can monitor biochemical indicators such as glucose, urea, and pH levels, alongside hemodynamic parameters like blood oxygen saturation and tumor markers. Additionally, integrating bioelectric signal monitoring^80^, including electrocardiograms (ECG), electroencephalograms (EEG), and electromyograms (EMG), provides comprehensive physiological data to support personalized diagnosis and treatment (Fig. 3d). Leveraging intelligent adjustment technologies, phototherapy devices can now dynamically optimize treatment parameters based on patients’ real-time conditions (Fig. 3e). Using multimodal deep learning algorithms^57^, these devices analyze physiological indicators, biochemical markers, and behavioral data to intelligently adjust parameters such as wavelength, light intensity, and exposure duration. This closed-loop system enhances phototherapy efficiency, reduces reliance on medical professionals, and facilitates home-based applications, paving the way for improved therapeutic outcomes and broader usability.

Based on cutting-edge research, we propose the aforementioned strategies from a clinical perspective to address these challenges in phototherapy. In the following sections, we will elaborate on the implementation of these strategies through advancements in multidisciplinary research and discuss their specific impact on enhancing the phototherapy application.

## Cutting-edge technologies and future processes

### 1. Advances in the development of LED phototherapy devices

To address the challenges of performance and applicability in LED phototherapy devices, researchers have introduced a variety of innovative strategies through material selection and process optimization. Significant progress has been made in areas such as soft substrates, soft active materials, soft emissive layers, soft encapsulations, and power supplies.

#### Soft substrate

**Fig. 4:**
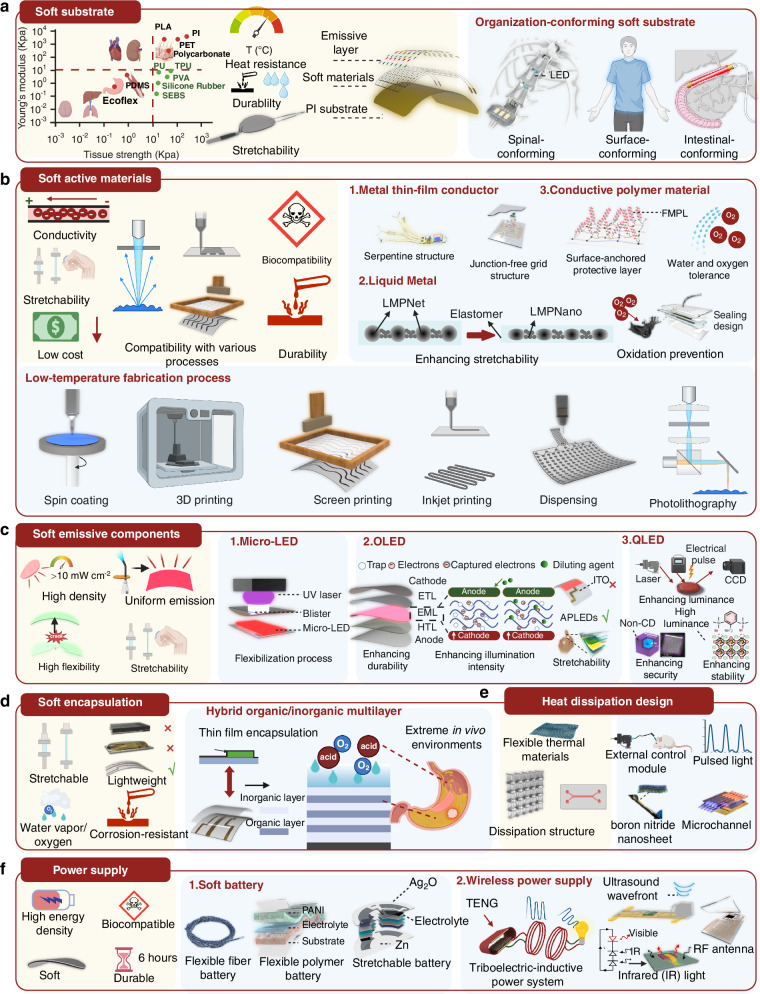
**Key progresses and innovations in processes and materials**. **a** The ideal properties required for soft substrates and representative applications in nervous system (reprinted with permission^52^. Copyright 2022, Springer Nature)^52^, body surface^13^, and gastrointestinal tract (reprinted with permission^43^ under Creative Common CC BY license. Copyright 2023, American Association for the Advancement of Science)^43^. **b** Soft active materials, including metal thin films (the left image is reprinted with permission^57^. Copyright 2023, Springer Nature. The right image is reprinted with permission^85^. Copyright 2021, John Wiley & Sons)^57,85^, liquid metals (reprinted with permission^86^. Copyright 2023, American Association for the Advancement of Science)^86,87,171^, and conductive polymer materials (reprinted with permission^89^. Copyright 2023, Springer Nature)^89^, along with their associated fabrication processes such as spin coating, 3D printing, screen printing, inkjet printing, dispensing, and photolithography. **c** Soft emissive component including Micro-LED^31^ and OLED (reprinted with permission^60^. Copyright 2022, Springer Nature)^60^ (reprinted with permission^105^. Copyright 2020, Springer Nature). **d** Soft encapsulation: hybrid organic/inorganic multilayer^69^. **e** Heat dissipation design: flexible thermal materials, external control module^72,73^, boron nitride nanoseed structure^74^, and microchannels^77^ for enhanced heat management. (reprinted with permission^74^ under Creative Common CC BY license. Copyright 2020, Springer Nature) **f** Power supply including soft battery (the middle image is reprinted with permission^122^ under Creative Common CC BY license. Copyright 2024, Springer Nature. The right image is reprinted with permission^148^. Copyright 2022, Springer Nature)^120–122^ wireless power supply^123–126^ (the image for RF antenna is reprinted with permission^77^. Copyright 2020, Springer Nature. The image for Infrared light is reprinted with permission^125^. Copyright 2018, National Academy of Sciences). Figure 4, created with BioRender.com, released under a Creative Commons Attribution-NonCommercial-NoDerivs 4.0 International license

The soft substrate is critical for determining the mechanical properties of phototherapy devices and ensuring compatibility with target tissues (Fig. 4a). An ideal substrate, as summarized in the table shown in Fig. 4a, should have a lower Young’s modulus than the target tissue while providing sufficient tensile strength, high-temperature resistance, chemical stability, and stretchability. Polyimide (PI) is well-suited for wearable and implantable devices due to its thermal and mechanical properties^17^, while softer, biocompatible materials like Polydimethylsiloxane (PDMS)^32^, Thermoplastic Polyurethane (TPU)^36^, Styrene-Ethylene-Butylene-Styrene^81^ (SEBS), and Ecoflex^82^ are preferable for dynamic areas. Tailoring substrate selection to specific applications enables optimal adaptation to target regions. For example, wrapping the spinal cord for optogenetic therapies in paralysis treatment^52^, conforming to the body surface for blue-light therapy in jaundice management^13^, or adhering to the intestinal lining for red-light therapy applications^43^ (Fig. 4a).

#### Soft active materials

In the fabrication of wearable or implantable phototherapy devices, soft active materials (Fig. 4b) should exhibit essential characteristics such as high electrical conductivity, stretchability, low cost, compatibility with diverse fabrication processes, biocompatibility, and durability. These requirements are designed to ensure that phototherapy devices maintain efficient optical power output to meet therapeutic needs while preserving stretchability.

Metal thin films are commonly used in device circuits due to their high conductivity and cost-effectiveness. They have been applied in optogenetic modulation for epilepsy^57^, phototherapeutic repair of cerebral infarction^55^, antimicrobial treatments for implant infections^83^, and red-light therapy for hair loss^18^. Structural optimization ensures their flexibility and mechanical stability. Current research mainly focuses on techniques such as serpentine patterns^57^, island-bridge structures^84^, or metal cracks^85^ to enhance their tensile strength. However, these approaches often significantly increase the device’s volume, which hinders miniaturization. Therefore, intrinsically stretchable materials such as liquid metals and polymers are better suited for future phototherapy devices. For instance, liquid metal materials, celebrated for their self-healing properties and exceptional ductility, are especially well-suited for dynamic applications^86^, such as treating arthritis, adapting to the beating surface of the heart^19,41^, and addressing urinary dysfunction through optogenetic modulation in response to bladder pressure changes^48^. Encapsulating liquid metals in elastomers can further improve their stretchability^87,88^. Furthermore, the liquid metal’s inherent chemical stability and the hermetic sealing design effectively address the challenges of oxidation, ensuring long-term performance and reliability in stretchable systems^86^. Conductive polymers, which offer good stretchability and low cost, are another promising option. Although they are susceptible to water and oxygen degradation, surface molecular modifications can significantly enhance their durability^89^, while maintaining good electrical conductivity (Fig. 4b). Many novel soft active materials can meet these requirements but are limited by their thermal tolerance, making them unsuitable for high-temperature processing. Consequently, the development of low-temperature fabrication techniques offers a pathway to expand the applications of these advanced materials. Cutting-edge processing methods currently include spin coating^90^, 3D printing^91^, screen printing^92^, inkjet printing^93^, dispensing printing, and photolithography^94^ (Fig. 4b).

#### Soft emissive components

The light sources used in flexible LED-based phototherapy devices can be classified into three main types: µLEDs, OLED, and QLEDs. µLEDs^31,52,95^ offer high light intensity ( ~ 1,000,000 nits), long lifespan ( ~ 100,000 h), and a narrow FWHM (15–20 nm), but have limitations in heat dissipation, flexibility, and light uniformity compared to OLED^13,25,43^ and QLED^61,96,97^. OLED-based devices require further enhancement in brightness and resistance to oxygen and moisture^98,99^, while QLEDs need to improve stability, ensuring no heavy metal incorporation^61,100^ (Table 2). To address these challenges, researchers have proposed various strategies.

**Table 2:** Characteristics and Comparison of LED-based Light Sources for Phototherapy Applications

	μ-LED	OLED	QLED (heavy-metal-free)	Refs
Light intensity	High ( ~ 1,000,000 nits)	Moderate ( ~ 10,000 nits achievable; EQE remains at 40% under 0–15,000 cd m^−^²)	High (> 300,000 nits)	^43,61,101,106,107^
Emission area	Point light source	Uniform surface-emitting light source	Uniform surface-emitting light source	^25,83,116^
FWHM	Narrow (15–20 nm)	Relatively broad (> 30 nm)	Narrow (20–30 nm)	^13,97^
Flexibility	None per se (relies on flexible substrate and chip transfer technology)	Soft and flexible	Soft and flexible	^31,60,96^
Life span	Long ( > 100,000 h)	Red/Green (Phosphorescent): Long (LT_50_ > ~100,000 hours); Blue (Fluorescent): Medium (LT_50_ ~ 10,000 hours); Blue (Phosphorescent): Limited but under improvement	Medium	^13,95,98,100,106,108,109^
Stability	High, oxygen- and humidity- resistant	Moderate, oxidation- and moisture-sensitive (requires excellent encapsulation)	Limited	^36,99,104,116,161^
Heat dissipation	Poor (spot high heat flow)	Excellent (uniform low heat flow)	Medium (faceted medium heat flow)	^13,31,97^

In the field of soft emission components, µLEDs/Micro-LED have become a focal point due to their high brightness, dense emission, and exceptional precision, making them the ideal selection for phototherapy device^101^ (Fig. 4c, Table 2). The challenge of transferring µLEDs onto flexible substrates^102^ has been effectively resolved with advanced laser transfer technologies^31^. With decreasing costs, flexible µLEDs show great promise as phototherapy light sources, particularly for complex surfaces like the face^18,31,34^, intestines^43^, brain^52,55^, heart^19^, and lungs^103^.

At the same time, OLED materials, conferring flexibility, low-temperature fabrication, and uniform light emission (Table 2), are already being applied in areas such as diabetic management^43^, hyperbilirubinemia treatment^13,38^, hemodynamic monitoring^60^, and neuroregulation^25^. However, their limited lifespan under high-intensity illumination constrains their broader use in phototherapy devices. While photobiomodulation and metronomic photodynamic therapy (PDT) can achieve therapeutic effects at lower light intensities, simultaneous improvements in brightness and durability are essential to extend OLED applications to a wider range of therapies, including photodynamic and photothermal treatments. Encouragingly, advances in thin-film packaging technologies are now effectively addressing these issues^104^. In parallel, new OLED optimization strategies, such as incorporating special solvents to dilute and reduce defects in the regions where electrons are captured within the OLED structure, along with the use of the double-sided polariton-enhanced Purcell effect to improve OLED stability, are further enhancing the light emission efficiency and lifespan of OLEDs^60,98,99,105–108^ (Fig. 4c). As detailed in Table 2, Red/Green (Phosphorescent) OLEDs exhibit lifespans exceeding ~100,000 h, while Blue OLEDs, especially the phosphorescent type, are showing ongoing improvements^108,109^. Remarkably, researchers have also achieved major breakthroughs in the fabrication of intrinsically stretchable OLEDs^60,105,110^, which currently represent the most stretchable light-emitting components developed to date.

QLEDs offer excellent flexibility, uniform surface emission, high light intensity ( > 300,000 nits), and narrow FWHM (20–30 nm)^111,112^ (Table 2). Current research focuses on developing biocompatible, heavy-metal-free (e.g., Cd-free) flexible QLEDs^61,100^. However, their limited stability and short lifespan remain significant challenges for phototherapy, with only a few studies addressing these issues^112–115^. New methods, such as electrically excited transient absorption (EETA)^61^, can effectively quantify the issues present in heavy-metal-free QLEDs. By optimizing the core-shell structure^100^ and surface passivation strategies^116^, it is possible to further enhance the luminous efficiency and stability, ensuring the biological safety of QLEDs (Fig. 4c).

#### Soft encapsulation

Encapsulation technologies also play a vital role in ensuring device stability. The ideal encapsulation should maintain the device’s stretchability, lightweight nature, and durability (Fig. 4d). Hybrid organic/inorganic multilayer encapsulation^104,117^ combines the gas-barrier properties of inorganic layers with the flexibility of organic layers, providing stability for phototherapy devices in extreme environments such as fluid-filled cavities like the abdominal and thoracic cavities, intracranial regions, and even the gastrointestinal tract, while minimizing the risk of toxic substance leakage—an essential consideration for implantable devices (Fig. 4d).

#### Heat dissipation design

LED phototherapy devices must address the potential side effects of heat generation during use. The skin’s outer layers, including the epidermis, dermis, and subcutaneous tissue, have low thermal conductivity (*κ* ≈ 0.3 W m^−1^ K^−1^)^118^, posing challenges for heat management. An ideal design integrates flexible thermal materials, efficient heat dissipation structures, and effective heat transfer mechanisms (Fig. 4e). In phototherapy, an effective strategy for preventing thermal damage is to transmit the LED light source *via* optical fibers while keeping the heat-generating control units external^119^. Metronomic PDT^72,73^, using low-dose, extended-duration, high-frequency light, reduces local thermal load accumulation and provides an effective heat management solution. This approach offers a viable strategy for addressing heat dissipation in PBM, optogenetics, and blue light therapy. However, further studies are needed to assess its applicability across other phototherapy strategies. Additionally, advanced thermal management designs, such as polymer/boron nitride nanosheets^74^ and miniaturized microchannel heat sinks^77^, offer potential solutions for enhancing heat dissipation in LED phototherapy devices (Fig. 4e).

#### Power supply

The power supply for phototherapy devices (Fig. 4f) plays a pivotal role in determining their functionality and application potential. An ideal power source should combine high energy density, soft, biocompatibility, and durability to meet the diverse demands of wearable and implantable phototherapy systems. Recent innovations in power systems have been driven by soft batteries and wireless power transfer technologies, enabling both device miniaturization and extended operational lifespans. Soft batteries have progressed significantly, making it possible to integrate them into textiles for wearable phototherapy devices, such as LED therapy patches designed to treat skin conditions like acne, wounds, and psoriasis^120^. Alternatively, they can be incorporated as miniaturized droplet^121^ or thin-film batteries^122^ in micro-sized phototherapy devices, allowing precise energy delivery for localized treatments. For implantable phototherapy devices, the choice of power source is dictated by the application. Solid-state batteries, with their ability to prevent toxic substance leakage, are better suited for long-term implantable systems. Wireless power transfer technologies, such as magnetic induction and triboelectric nanogenerators^123^ (TENGs), can harness kinetic energy from the human body to power implantable devices. These technologies expand the usability of phototherapy systems in scenarios requiring portability and frequent use. Additional methods, such as ultrasonic power^124^, infrared functionality^125^, and far-field communication (RF)^126^, present promising wireless energy options for implantable applications. These approaches are particularly advantageous for treatments requiring minimal device maintenance and long-term functionality. Despite these advances, phototherapy devices often demand substantial power for high-intensity light output, particularly in applications such as deep tissue treatment^58^ or PDT^127^. In these cases, wireless power solutions alone are insufficient to sustain real-time energy needs and they must be paired with integrated batteries to provide reliable energy storage. The careful selection and integration of power systems, tailored to the specific clinical context, ensures that phototherapy devices achieve optimal performance across a wide range of medical applications.

### 2. Future manufacturing processes for LED phototherapy devices

In response to the challenges currently faced by LED-based phototherapy devices in clinical applications, as well as recent advancements in the field, we present a universal fabrication process for future wearable/implantable LED phototherapy devices.

#### Selection of soft substrates

**Fig. 5:**
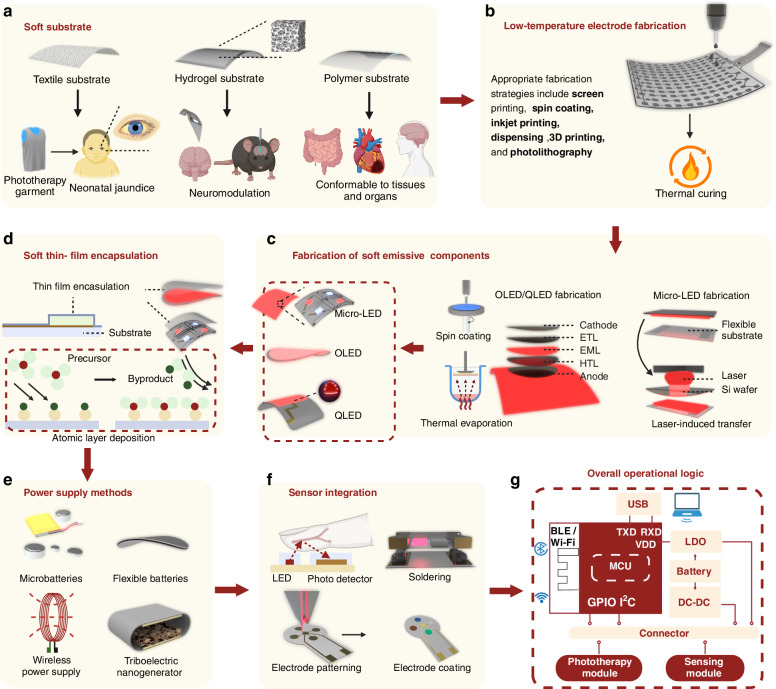
**Technical roadmap for integrating monitoring and treatment in future phototherapy devices.** **a** Selecting appropriate soft substrates based on specific clinical applications (e.g., textile^13,34^, hydrogel^70^, polymer^36^). **b** Fabrication of the conductive layer utilizing low-temperature processing techniques^90,93,172^. **c** Fabrication methods for soft emissive component: laser-induced transfer for micro-LED^31,62,133^, spin coating^60^ or thermal evaporation^173^ for OLED. **d** Soft thin-film encapsulation using atomic layer deposition^135^. **e** Novel power supply methods: microbatteries^71^, flexible batteries^120^, wireless power supply^174^, and triboelectric nanogenerator^123^. **f** Feasible strategies for integrating phototherapy devices with sensors^79,140^. **g** Overall operational logic of future phototherapy devices. Figure 5, created with BioRender.com, released under a Creative Commons Attribution-NonCommercial-NoDerivs 4.0 International license

Selecting the appropriate soft substrate based on the intended application is a key step toward achieving device flexibility and stretchability **(**Fig. 5a**)**. Textile-based substrates, with their breathability and softness, are particularly suitable for large-area treatment scenarios^13^, such as phototherapy garments for jaundice treatment, phototherapy knee braces for arthritis management, and phototherapy caps for promoting hair growth. Hydrogel substrates, known for their high biocompatibility and transparency, are preferred for devices in direct contact with the skin or organs^70^. Polymer substrates, which balance mechanical strength and flexibility, are better suited for highly deformable regions and implantable phototherapy devices. Current soft substrates face significant challenges in conforming to complex biological surfaces (e.g., brain gyri or joint folds). As shown in Fig. 4a (left), the elastic modulus of brain and lung tissues is an order of magnitude lower than that of common polymer substrates (PI, PET, PDMS, SEBS), and the grooves in regions like the brain and skin hinder full adhesion of flexible phototherapy devices, affecting treatment uniformity^128^. Flexibility is influenced by device thickness, Young’s modulus, and width^66^, with studies showing that a thickness of 10–100 μm ensures effective brain tissue adaptation^129^. Ultra-flexible nanoelectronics (<10 μm feature size, 1 μm thickness) can further reduce chronic inflammation^130^. Currently, phototherapy devices are often fabricated on polymer substrates, and researchers have employed various methods to optimize the interface compatibility with tissues. OLED and QLED devices have achieved thicknesses around 10 μm^25,43,100^, offering excellent flexibility, while micro-LEDs can enhance tissue and organ compatibility and phototherapy efficacy through substrate design (octopus’ structure)^95^.

#### Fabrication processes for electrodes

To address the temperature tolerance of new materials, low-temperature fabrication techniques are better suited for future LED phototherapy devices^131^ (Fig. 5b). Methods like inkjet and 3D printing enable precise fabrication of complex structures^93^ while optimizing conductive ink properties for multilayer flexible circuits. Spin-coating^132^ further improves the uniformity and optical performance of emissive layers with precise film thickness control (Fig. 5b).

#### Preparation of soft emissive components

The manufacturing processes for soft emission components are continuously optimized to improve efficiency and reduce costs (Fig. 5c). Laser-induced transfer technology enables high-precision and large-scale production of µLEDs^133^, making them one of the most promising solutions for future phototherapy applications. Meanwhile, thermal evaporation^132^ techniques and advanced spin-coating^90^ methods significantly enhance the luminous efficiency and lifespan of OLED and QLED devices by improving the fabrication of the emissive layer. When combined with elastomers, OLED^60^ and QLED^134^ devices exhibit enhanced stretchability and flexibility. Both of these light-emitting components can achieve ultra-uniform surface emission and meet the optical power requirements of phototherapy. Additionally, Surface-Mount Device LEDs can be directly integrated into phototherapy devices through soldering^36^ (Fig. 5c).

#### Soft thin-film encapsulation

To preserve the softness and thinness of future LED phototherapy devices, atomic layer deposition^135^ (ALD) (Fig. 5d) can sequentially deposit organic and inorganic layers, providing effective water and oxygen barriers. ALD’s self-limiting growth mechanism ensures smooth atomic surfaces and uniform nanoscale films, making it a promising approach for thin-film encapsulation^69^.

#### Energy supply methods

The power supply for future LED phototherapy devices should be tailored to specific application requirements (Fig. 5e). For wearable devices, rechargeable micro-batteries are ideal, offering extended use^36^ for applications such as acne, wounds, and psoriasis. Implantable devices for short-term applications may utilize high-energy-density micro-batteries or soft batteries, particularly for treating heart diseases, cancer, and deep tissue disorders. Long-term implantable devices require wireless power transfer systems to provide sustained energy supply. While wireless coils are commonly used, their size constraints limit achievable power levels and application scenarios. Alternative methods, including RF communication^126^, ultrasound^124^, infrared^125^, and energy harvesting technologies like piezoelectric nanogenerators^136^ or self-powered systems^137^, show potential but are insufficient for high-power phototherapy applications. However, their real-time power output often falls short of the demands of high-intensity phototherapy applications, necessitating battery storage to ensure a reliable and continuous energy supply.

#### Integration of sensing modules

The integration of sensors is pivotal for enhancing the intelligence of phototherapy devices (Fig. 5f). Electrodes for electrochemical and electrophysiological sensors are typically patterned using laser engraving^138^, with functional materials deposited to enable targeted data collection^139^. Photoelectric detectors, based on spectroscopic principles, are fabricated similarly to LED emissive materials^68^, while pre-packaged detectors can be soldered directly onto flexible circuits^140^. As shown in Fig. 5g, the design of future phototherapy devices incorporates micro-processing chips that collect sensor data and use built-in algorithms to dynamically adjust treatment parameters in real time. Sensors monitoring parameters such as tissue oxygen levels, skin temperature, and light absorption enable real-time feedback and optimization of therapy, ensuring maximum efficacy tailored to specific clinical conditions.

Integrating sensors that monitor physiological parameters marks a significant advancement in phototherapy device technology. These sensors enable real-time feedback and dynamic optimization of treatment parameters, ensuring maximum therapeutic efficacy by adapting to specific clinical conditions. Seamless integration of sensors during device fabrication enhances functionality, streamlines design, and creates compact, efficient systems that reduce manual adjustments and improve adaptability across diverse clinical scenarios. Future devices, leveraging advanced Internet of Things technologies, will better meet clinical needs and address the growing demand for professional phototherapy services in home settings, further broadening the scope and impact of phototherapy technology.

## Future directions: towards intelligent and integrated phototherapy systems

**Fig. 6:**
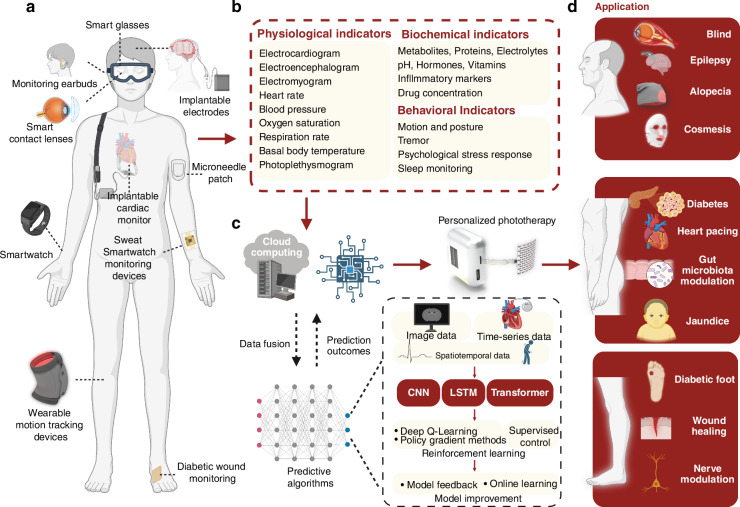
**Wearable monitoring devices and potential algorithms**. **a** Existing wearable monitoring devices: smart glasses^141^, contact lenses^142,143^, earbuds^80^, implantable electrodes^57^, microneedle patch^79^, smartwatch^152^, sweat monitoring devices^164^, wearable motion tracking devices^63^, diabetic wound monitoring^138^. **b** Monitoring indicators of wearable devices, including physiological indicators, biochemical indicators and behavioral indicators. **c** Workflow for processing monitoring data and potential algorithms, including acquiring monitoring information from sensors, data fusion, training models to predict patient physiological conditions, and outputting personalized phototherapy parameters. **d** Applications combining wearable monitoring devices with phototherapy devices^13,17,18,31,36,41,46,48,52,175^. Figure 6, created with BioRender.com, released under a Creative Commons Attribution-NonCommercial-NoDerivs 4.0 International license

The rapid development of soft wearable and implantable sensors drives phototherapy devices toward enhanced intelligence and integration. A range of wearable and implantable sensor devices (Fig. 6a), including smart glasses^141^, smart contact lenses^142–144^, monitoring headphones^80^, implantable EMG, EEG and ECG devices^57^, smartwatches^141^, microneedle patches^79^, tendon sensors^63^, and wound monitoring patches^138^, have been developed to continuously monitor a wide variety of physiological, biochemical, and behavioral indicators (Fig. 6b).

In the treatment of chronically infected wounds **(**Fig. 6c**)**, phototherapy devices integrated with sensors collect key physiological and biochemical data, such as pH, temperature, lactate, uric acid, and oxygen saturation, providing real-time insights into wound infection and healing progress^138^ **(**Fig. 6b**)**. These data are wirelessly transmitted to cloud platforms, where deep learning models, trained on large datasets, analyze the wound state, and recommend optimized phototherapy parameters **(**Fig. 6c**)**. Models are embedded into phototherapy devices, using algorithms like CNNs for image data, LSTMs for time-series data, and Transformers for multimodal spatiotemporal inputs. Reinforcement learning methods, including Deep Q-Learning, Policy Gradient methods, and supervised control, refine the settings by learning from therapeutic outcomes. Model feedback and online learning further enhance adaptability, ensuring continuous optimization based on real-world data. This intelligent system dynamically adjusts phototherapy parameters—such as wavelength, intensity, and duration—creating a closed-loop framework for precise, efficient, and personalized wound management **(**Fig. 6c**)**.

Based on the integration strategy of phototherapy devices with AI, Table 3 summarizes representative sensors that could be integrated with phototherapy systems in the future. Possible application scenarios are illustrated in Fig. 6d, with their integration approaches described as follows:

**Table 3:** Summary of Sensor Modules Compatible with Phototherapy Devices

Application site	Targeted disease	Application method	Detection indicators	Detection mechanism	Highlights	Phototherapy strategy	Refs
Head	Epilepsy	Implantation	Oxygenation in deep tissue	Spectroscopy	Ultra-flexible film OLEDs provide precise light stimulation to the brain and can conform to various anatomical structures.	Use of blue/yellow wavelength light to promote neuroregeneration and seizure prevention, with intensity modulated based on oxygenation and EEG signal feedback.	^67^
Postoperative free flaps and replanted digits	Vascular compromise	Noninvasive, on-skin biosensor	Blood oxygen saturation, pulse rate	Photoplethysmography (PPG) signals	Wireless, continuous monitoring with a self-adhesive robust substrate	Use of near-infrared light to improve vascular perfusion and prevent tissue necrosis, with real-time modulation based on blood oxygen saturation and pulse rate data.	^140^
Tendon/Connective tissue	Biomechanical strain monitoring	Implantable sensor	Strain in connective tissue	Capacitive strain sensing (wireless)	Stretchable, wireless operation, high sensitivity, biocompatible	Use of near-infrared or targeted wavelength light to promote tissue repair and regeneration, with intensity modulated by strain data feedback	^63^
Brain	Postoperative monitoring of intracranial pressure and temperature	Implantation	Intracranial pressure, temperature	Pressure and temperature sensors	Fully bioresorbable, eliminating the need for surgical removal; demonstrated efficacy in rat models	Use of targeted wavelength light (e.g., near-infrared) to promote neuroregeneration and reduce inflammation based on detected brain pressure or temperature abnormalities	^162^
Bladder	Bladder dysfunction	Implantation	Bladder volume and voiding events	Soft, stretchable strain gauge to monitor bladder filling and voiding	Fully implantable, wireless system combining real-time monitoring with optogenetic neuromodulation; demonstrated efficacy in normalizing bladder function in rat models	Use of optogenetic stimulation to modulate bladder sensory afferents, thereby controlling bladder function	^48^
Ear	Neurological conditions, metabolic disorders	Non-invasive, in-ear wearable device	Brain activity (EEG, EOG, EDA), lactate levels in sweat	EEG, EOG, EDA, electrochemical sensing	Flexible, integrated sensor array for simultaneous monitoring of electrophysiological signals and sweat biomarkers; potential for real-time health monitoring	Use of targeted blue and near-infrared light to improve neurological function and metabolic regulation, guided by real-time EEG and lactate level monitoring.	^80^
Skin (Wrist)	Metabolic diseases (e.g., diabetes)	Noninvasive, on-skin wearable microneedle array	Glucose, lactate, alcohol levels	Electrochemical sensing	Integrated microneedle array enabling wireless, real-time, continuous monitoring of multiple biomarkers; paired with a smartphone app for data visualization	Use of near-infrared light for metabolic stimulation, with real-time intensity adjustments based on glucose, lactate, and alcohol level feedback.	^79^
Skin (e.g., forearm)	Metabolic monitoring	Noninvasive, on-skin wearable biosensor	Glucose, pH, sodium ions, sweat rate, skin temperature	Electrochemical detection, impedance analysis	Autonomous operation powered by flexible perovskite solar cell; continuous monitoring under lighting conditions; wireless data transmission	Use of near-infrared and green light to enhance metabolic processes, dynamically modulated by sweat biomarkers and skin temperature data.	^78^
Neck (carotid artery and jugular vein)	Cardiovascular health monitoring	Noninvasive, on-skin conformal ultrasonic device	Central blood pressure waveforms	Ultrasonic sensing	Ultrathin (240 μm), stretchable (up to 60%) device enabling continuous and accurate monitoring of deep arterial and venous sites; overcomes limitations of traditional bulky ultrasound probes	Use of targeted wavelength light to improve vascular elasticity and function, with intensity modulated by central blood pressure waveform feedback.	^163^
Skin (e.g., forearm)	Stress response assessment	Noninvasive, on-skin electronic skin	Vital signs: pulse waveform, galvanic skin response, skin temperature; Sweat biomarkers: glucose, lactate, uric acid, sodium ions, potassium ions, ammonium	Electrochemical sensors for sweat biomarkers; Physical sensors for vital signs	Continuous monitoring of physiological and biochemical stress markers; High accuracy in differentiating stressors with machine learning integration	Use of blue and red light to alleviate physiological stress markers, dynamically adjusted by sweat biomarker and vital sign data processed through machine learning.	^164^
Chest and foot	Vital signs monitoring in neonates	Noninvasive, on-skin epidermal electronic systems	ECG, (PPG), skin temperature	ECG: electrical potential measurement; PPG: optical sensing of blood volume changes; temperature: thermal sensing	Wireless, battery-free operation; ultrathin, soft, skin-like devices; real-time, in-sensor data analytics; time-synchronized, continuous data streaming; gentle adhesive interfaces suitable for fragile neonatal skin; compatibility with medical imaging techniques	Use of blue light for jaundice treatment and oxygenation improvement, integrated with vital sign data from ECG and PPG sensors for optimized therapy delivery.	^154^
Forehead	Neonatal jaundice	Noninvasive, wearable transcutaneous bilirubinometer	Bilirubin levels, blood oxygen saturation (SpO₂), heart rate (HR)	Colorimetric analysis for bilirubin; pulse oximetry for SpO₂ and HR	Continuous, real-time monitoring; simultaneous measurement of bilirubin, SpO₂, and HR; effective during phototherapy	Automatic adjustment of blue light intensity for bilirubin breakdown, based on real-time bilirubin, SpO₂, and heart rate monitoring.	^155^
Skin surface (e.g., arm)	Monitoring of hemodynamic parameters and metabolic biomarkers	Non-invasive, skin-conformal wearable patch	Blood pressure, heart rate, glucose, lactate, caffeine, alcohol levels	Ultrasonic transducers for hemodynamic parameters; electrochemical sensors for metabolic biomarkers	Simultaneous monitoring of multiple parameters; flexible and stretchable design; real-time data acquisition	Use of near-infrared light for hemodynamic improvement, dynamically controlled by blood pressure and metabolic biomarker feedback.	^151^
Skin surface over chronic wounds	Infected chronic wounds	Stretchable, wireless, wearable, bioelectronic patch	Uric acid, lactate, pH, temperature	Multiplexed multimodal electrochemical biosensor array	Continuous, real-time monitoring; controlled drug delivery; electrical stimulation for tissue regeneration	Use of antimicrobial blue light therapy and tissue-regenerative near-infrared light, with controlled intensity and duration based on pH, uric acid, and temperature feedback.	^138^
Skin surface (e.g., forearm)	Monitoring of sweat biomarkers	Noninvasive, stretchable, on-skin biosensor	pH, sodium ions, glucose, lactate	Electrochemical sensors	Integrated printed battery; real-time data visualization via electrochromic display; fully autonomous operation	Use of light-based therapy for metabolic enhancement, with real-time adjustments guided by sweat biomarker data visualized on electrochromic display.	^148^
Skin surface (e.g., chest, abdomen)	Continuous monitoring of vital signs in neonates and pediatric patients	Noninvasive, skin-conformal, wireless biosensor	Heart rate, respiration rate, temperature, blood oxygenation	Soft, flexible sensors with wireless data transmission	Measurement equivalency to clinical standards; additional features like movement tracking and acoustic cardiac monitoring; supports skin-to-skin contact	Use of therapeutic light for respiratory and circulatory support, with intensity modulated by real-time heart rate, respiration rate, and oxygenation data.	^153^

For the head, phototherapy devices based on optogenetics have been developed for the treatment of epilepsy^57^, Alzheimer’s disease^145,146^, and depression^147^ (Figs. 1a, 6d**)**. These devices can integrate with wearable sensors, such as earbuds^80^ and smart contact lenses^142,143^, to monitor brainwaves, neural signals, and tissue oxygenation levels (Table 3), enabling personalized optical therapies tailored to the patient’s disease progression. Additionally, facial sweat sensors^148^ and electrochemical biosensors^79^ (Table 3) have the potential to be combined with phototherapy masks^31^ and soft light-emitting patch^17^ for applications in acne treatment^149^, pigmentation disorders^150^, and facial rejuvenation^62^ (Figs. 1a, 6d).

In the torso region, wearable microneedle sensors^79^, electrochemical sweat sensors^151^, and smartwatches^152^ are capable of monitoring blood glucose levels, blood oxygen saturation, heart rate, pulse, ECG signals, and physical activity acceleration, providing valuable insights into both cardiac and behavioral functions (Table 3). These advancements support applications such as light-based modulation of glucose metabolism^43^, Parkinson^49^ and optogenetic cardiac pacemakers^41^(Figs. 1a, 6d). Moreover, integrating transcutaneous bilirubin sensors^153–155^ with phototherapy garments^13^ enhances the treatment of hyperbilirubinemia (Table 3), while implantable sensors combined with phototherapy devices hold promise for simultaneously monitoring gastrointestinal microbiota and modulating gut flora^43^ (Fig. 6d).

For the limbs, sensors that measure deep tissue oxygen concentration^67^ and peripheral metabolites^79^ (Table 3), when integrated with phototherapy devices, enable closed-loop management of chronic infectious wounds, offering new solutions for addressing diabetes-related complications(Fig. 6d). Furthermore, optogenetic peripheral nerve modulation is becoming increasingly sophisticated. For example, integrating implantable pressure sensors with phototherapy devices allows precise regulation of bladder pressure, providing a solution for urinary dysfunction^48^. Lastly, incorporating sensors capable of detecting visual evoked potentials and electroretinography signals into phototherapy glasses allows real-time monitoring of visual function and supports vision restoration through targeted phototherapy^37^(Fig. 6d).

The future of intelligent phototherapy systems extends beyond parameter adjustment to deep integration with treatment feedback mechanisms. Through real-time data analysis, these systems can detect subtle changes in disease states and automatically update AI models, further enhancing treatment precision. The high level of integration between phototherapy devices and sensors enables lightweight and non-invasive designs, making them highly suitable for daily use in home settings.

In conclusion, the advancements in soft wearable and implantable sensors, combined with the powerful data processing capabilities of AI algorithms, provide a solid foundation for the widespread application of intelligent phototherapy devices. This direction not only enhances treatment efficiency and safety but also advances the field of precision medicine, offering personalized solutions for a range of complex diseases.

## Conclusions and future perspective

This review adopts the perspective of clinical phototherapy practitioners, addresses specific clinical needs, and summarizes the extensive applications of phototherapy devices in the medical field. It highlights the major challenges encountered during the implementation of wearable and implantable phototherapy devices. Drawing on advancements in photomedicine, materials science, and soft electronics, the review proposes targeted strategies to address these challenges, summarizes interdisciplinary research contributing to the future development of phototherapy devices, and outlines a potential fabrication roadmap for future devices. Furthermore, it explores the integration of emerging AI technologies and soft sensing modules to design closed-loop phototherapy-monitoring systems for enhanced therapeutic outcomes.

Building on the outlined challenges and strategies for advancing phototherapy devices, it is essential to recognize the transformative role of photomedicine in clinical practice. Photomedicine has emerged as a cornerstone in disease diagnosis and treatment, transitioning from its historical role as an adjunct therapy to serving as a first-line treatment for various conditions. It has demonstrated remarkable efficacy in managing neonatal jaundice, wound healing, pigmentary disorders, and vascular anomalies. This transition underscores its value as a low-cost, highly precisive physical therapy. The increasing clinical relevance of photomedicine necessitates its broader adoption, particularly as advancements in technology continue to improve its therapeutic precision and accessibility. Despite its promise, significant challenges remain in the design and engineering of wearable and implantable phototherapy devices. Wearable devices often face issues related to portability, personalization, illumination uniformity, and compatibility with the mechanical and optical properties of target tissues. We highlight the potential of OLED and µLED technologies, combined with optical lenses and diffusion layers, to improve illumination uniformity and facilitate miniaturization through modular designs. On the other hand, implantable devices, while offering more targeted treatment, encounter limitations such as insufficient energy supply, reduced durability, encapsulation leakage, and biosafety concerns. Novel biomaterials, multilayer thin-film encapsulation, and soft or micro-batteries can be incorporated to enhance durability and biosafety. Additionally, integrating sensors such as electrochemical, spectroscopic, and electrophysiological systems enable closed-loop diagnostic and therapeutic capabilities, further advancing the precision and effectiveness of phototherapy applications.

Advances in light-emitting and conductive materials are also shaping the future of phototherapy devices. µLEDs and OLEDs are identified as the most suitable light-emitting materials due to their high efficiency, flexibility, and biocompatibility. Similarly, liquid metals and stretchable conductive polymers are emerging as promising candidates for soft active materials, enabling devices to better conform to complex anatomical surfaces. To realize the potential of these technologies, low-temperature fabrication techniques are essential, particularly for new soft active materials that are sensitive to high-temperature environments. Additionally, hybrid organic-inorganic thin-film encapsulation and advanced wearable/implantable power solutions further accelerate the development of future phototherapy devices. To support the development of next-generation phototherapy devices, we outline a comprehensive design framework that incorporates the selection of soft substrates, active material deposition, and device schematics. This framework provides a roadmap for researchers and engineers to create devices that meet the stringent requirements of medical applications, including safety, durability, and user-friendliness.

Finally, integrating AI with phototherapy devices represents a significant opportunity to advance the field. Leveraging wearable and implantable sensing technologies, AI has the potential to enable closed-loop systems that combine diagnostic and therapeutic capabilities, allowing for real-time adjustments to various clinical applications. Such intelligent systems hold transformative potential, as evidenced by proposed application scenarios that demonstrate their ability to improve outcomes across a range of medical conditions. These advancements highlight the exciting future of photomedicine and its growing role in precision healthcare.

## References

[1] Li, X. S. et al. Clinical development and potential of photothermal and photodynamic therapies for cancer. *Nat. Rev. Clin. Oncol.* **17**, 657–674 (2020). 32699309 10.1038/s41571-020-0410-2

[2] Finsen, N. R. *Phototherapy* (Edward Arnold Publishers Ltd., 1901).

[3] Maiman, T. H. Stimulated optical radiation in ruby. *Nature* **187**, 493–494 (1960).

[4] Batta, K. et al. Randomised controlled study of early pulsed dye laser treatment of uncomplicated childhood haemangiomas: results of a 1-year analysis. *Lancet* **360**, 521–527 (2002). 12241656 10.1016/S0140-6736(02)09741-6

[5] Dolmans, D. E. J. G. J., Fukumura, D. & Jain, R. K. Photodynamic therapy for cancer. *Nat. Rev. Cancer* **3**, 380–387 (2003). 12724736 10.1038/nrc1071

[6] Seaton, E. D. et al. Pulsed-dye laser treatment for inflammatory acne vulgaris: randomised controlled trial. *Lancet* **362**, 1347–1352 (2003). 14585635 10.1016/s0140-6736(03)14629-6

[7] Lu, M. et al. Bacteria-specific phototoxic reactions triggered by blue light and phytochemical carvacrol. *Sci. Transl. Med.* **13**, eaba3571 (2021). 33408183 10.1126/scitranslmed.aba3571

[8] Kim, T. I. et al. Refractive surgery. *Lancet* **393**, 2085–2098 (2019). 31106754 10.1016/S0140-6736(18)33209-4

[9] Anderson, R. R. & Parrish, J. A. Selective photothermolysis: precise microsurgery by selective absorption of pulsed radiation. *Science* **220**, 524–527 (1983). 6836297 10.1126/science.6836297

[10] Parker, T. et al. Gamma knife radiosurgery for uveal melanomas and metastases: a systematic review and meta-analysis. *Lancet Oncol.* **21**, 1526–1536 (2020). 33152286 10.1016/S1470-2045(20)30459-9

[11] Ho, P. W. L., Szeto, C. C. & Chow, K. M. Continuous glucose monitoring device causes consternation on chest x-ray. *Lancet* **399**, 2412 (2022). 35753341 10.1016/S0140-6736(22)01017-0

[12] An, L. Y. et al. Sexual dimorphism in melanocyte stem cell behavior reveals combinational therapeutic strategies for cutaneous repigmentation. *Nat. Commun.* **15**, 796 (2024). 38280858 10.1038/s41467-024-45034-3 PMC10821900

[13] Choi, S. et al. Wearable photomedicine for neonatal jaundice treatment using blue organic light-emitting diodes (OLEDs): toward textile-based wearable phototherapeutics. *Adv. Sci.* **9**, 2204622 (2022). 10.1002/advs.202204622 PMC9762290 36310107

[14] Stitt, A. W. et al. The progress in understanding and treatment of diabetic retinopathy. *Prog. Retinal Eye Res.* **51**, 156–186 (2016). 10.1016/j.preteyeres.2015.08.001 26297071

[15] Tang, Y. L. et al. Green light analgesia in mice is mediated by visual activation of enkephalinergic neurons in the ventrolateral geniculate nucleus. *Sci. Transl. Med.* **14**, eabq6474 (2022). 36475906 10.1126/scitranslmed.abq6474

[16] Oh, P. S. & Jeong, H. J. Therapeutic application of light emitting diode: Photo-oncomic approach. *J. Photochem. Photobiol. B: Biol.* **192**, 1–7 (2019). 10.1016/j.jphotobiol.2019.01.003 30654264

[17] Lee, S. Y. et al. Combinatorial wound healing therapy using adhesive nanofibrous membrane equipped with wearable LED patches for photobiomodulation. *Sci. Adv.* **8**, eabn1646 (2022). 35427152 10.1126/sciadv.abn1646 PMC9012471

[18] Lee, H. E. et al. Trichogenic photostimulation using monolithic flexible vertical AlGaInP light-emitting diodes. *ACS Nano* **12**, 9587–9595 (2018). 30125485 10.1021/acsnano.8b05568

[19] Deng, K. C. et al. A biodegradable, flexible photonic patch for in vivo phototherapy. *Nat. Commun.* **14**, 3069 (2023). 37244895 10.1038/s41467-023-38554-x PMC10224912

[20] Sakai, J. Functional near-infrared spectroscopy reveals brain activity on the move. *Proc. Natl. Acad. Sci. USA* **119**, e2208729119 (2022). 35709323 10.1073/pnas.2208729119 PMC9231602

[21] Tomassetti, C. et al. Estimation of the endometriosis fertility index prior to operative laparoscopy. *Hum. Reprod.* **36**, 636–646 (2021). 33367865 10.1093/humrep/deaa346

[22] Chen, X. et al. Far infrared irradiation suppresses experimental arthritis in rats by down-regulation of genes involved inflammatory response and autoimmunity. *J. Adv. Res.* **38**, 107–118 (2022). 35572409 10.1016/j.jare.2021.08.015 PMC9091720

[23] Lee, H. E. et al. Optogenetic brain neuromodulation by stray magnetic field via flash-enhanced magneto-mechano-triboelectric nanogenerator. *Nano Energy* **75**, 104951 (2020).

[24] Rajalingham, R. et al. Chronically implantable LED arrays for behavioral optogenetics in primates. *Nat. Methods* **18**, 1112–1116 (2021). 34462591 10.1038/s41592-021-01238-9

[25] Kim, D. et al. Ultraflexible organic light-emitting diodes for optogenetic nerve stimulation. *Proc. Natl. Acad. Sci. USA* **117**, 21138–21146 (2020). 32817422 10.1073/pnas.2007395117 PMC7474697

[26] Van Tran, V. et al. Light emitting diodes technology-based photobiomodulation therapy (PBMT) for dermatology and aesthetics: recent applications, challenges, and perspectives. *Opt. Laser Technol.* **135**, 106698 (2021).

[27] Yang, L. D. et al. Mitochondria as a target for neuroprotection: role of methylene blue and photobiomodulation. *Transl. Neurodegeneration* **9**, 19 (2020). 10.1186/s40035-020-00197-z PMC7262767 32475349

[28] Kim, W. S. et al. AI-enabled, implantable, multichannel wireless telemetry for photodynamic therapy. *Nat. Commun.* **13**, 2178 (2022). 35449140 10.1038/s41467-022-29878-1 PMC9023557

[29] Piksa, M. et al. The role of the light source in antimicrobial photodynamic therapy. *Chem. Soc. Rev.* **52**, 1697–1722 (2023). 36779328 10.1039/d0cs01051k

[30] Pham, T. C. et al. Recent strategies to develop innovative photosensitizers for enhanced photodynamic therapy. *Chem. Rev.* **121**, 13454–13619 (2021). 34582186 10.1021/acs.chemrev.1c00381

[31] Kim, M. S. et al. Clinical validation of face-fit surface-lighting micro light-emitting diode mask for skin anti-aging treatment. *Adv. Mater.* **36**, 2411651 (2024). 10.1002/adma.202411651 39439130

[32] Zhang, H. et al. Biocompatible light guide-assisted wearable devices for enhanced UV light delivery in deep skin. *Adv. Funct. Mater.* **31**, 2100576 (2021).

[33] Bachelez, H. et al. Tofacitinib versus etanercept or placebo in moderate-to-severe chronic plaque psoriasis: a phase 3 randomised non-inferiority trial. *Lancet* **386**, 552–561 (2015). 26051365 10.1016/S0140-6736(14)62113-9

[34] Jeon, Y. et al. Sandwich-structure transferable free-form OLEDs for wearable and disposable skin wound photomedicine. *Light Sci. Appl.* **8**, 114 (2019). 31839934 10.1038/s41377-019-0221-3 PMC6900403

[35] Yang, L. et al. Biofilm microenvironment triggered self-enhancing photodynamic immunomodulatory microneedle for diabetic wound therapy. *Nat. Commun.* **14**, 7658 (2023). 37996471 10.1038/s41467-023-43067-8 PMC10667311

[36] Li, M. et al. A wearable and stretchable dual-wavelength LED device for home care of chronic infected wounds. *Nat. Commun.* **15**, 9380 (2024). 39477919 10.1038/s41467-024-53579-6 PMC11525593

[37] Sahel, J. A. et al. Partial recovery of visual function in a blind patient after optogenetic therapy. *Nat. Med.* **27**, 1223–1229 (2021). 34031601 10.1038/s41591-021-01351-4

[38] McDonagh, A. F. Letter: phototherapy and hyperbilirubinaemia. *Lancet* **1**, 339 (1975). 10.1016/s0140-6736(75)91252-0 46484

[39] Figueiro Longo, M. G. et al. Effect of transcranial low-level light therapy vs sham therapy among patients with moderate traumatic brain injury: a randomized clinical trial. *JAMA Netw. Open* **3**, e2017337 (2020). 32926117 10.1001/jamanetworkopen.2020.17337 PMC7490644

[40] Kim, J. et al. Implantable MicroLED-mediated chemo-photodynamic combination therapy for glioma treatment. *Adv. Funct. Mater.* **34**, 2316386 (2024).

[41] Hsueh, B. et al. Cardiogenic control of affective behavioural state. *Nature* **615**, 292–299 (2023). 36859543 10.1038/s41586-023-05748-8 PMC9995271

[42] Ausra, J. et al. Wireless, fully implantable cardiac stimulation and recording with on-device computation for closed-loop pacing and defibrillation. *Sci. Adv.* **8**, eabq7469 (2022). 36288311 10.1126/sciadv.abq7469 PMC9604544

[43] Sim, J. H. et al. OLED catheters for inner-body phototherapy: a case of type 2 diabetes mellitus improved via duodenal photobiomodulation. *Sci. Adv.* **9**, eadh8619 (2023). 37656783 10.1126/sciadv.adh8619 PMC10854432

[44] Qiao, L. L. et al. A sensitive red/far-red photoswitch for controllable gene therapy in mouse models of metabolic diseases. *Nat. Commun.* **15**, 10310 (2024). 39604418 10.1038/s41467-024-54781-2 PMC11603164

[45] Shao, J. W. et al. Smartphone-controlled optogenetically engineered cells enable semiautomatic glucose homeostasis in diabetic mice. *Sci. Transl. Med.* **9**, eaal2298 (2017). 28446682 10.1126/scitranslmed.aal2298

[46] Kawana, Y. et al. Optogenetic stimulation of vagal nerves for enhanced glucose-stimulated insulin secretion and β cell proliferation. *Nat. Biomed. Eng.* **8**, 808–822 (2024). 37945752 10.1038/s41551-023-01113-2 PMC11310082

[47] Mansouri, M. et al. Smart-watch-programmed green-light-operated percutaneous control of therapeutic transgenes. *Nat. Commun.* **12**, 3388 (2021). 34099676 10.1038/s41467-021-23572-4 PMC8184832

[48] Mickle, A. D. et al. A wireless closed-loop system for optogenetic peripheral neuromodulation. *Nature* **565**, 361–365 (2019). 30602791 10.1038/s41586-018-0823-6 PMC6336505

[49] Sinha, G. Trials begin for a new weapon against Parkinson’s: light. *Science* **369**, 1415–1416 (2020). 32943504 10.1126/science.369.6510.1415

[50] Li, D. Y. et al. Photostimulation of brain lymphatics in male newborn and adult rodents for therapy of intraventricular hemorrhage. *Nat. Commun.* **14**, 6104 (2023). 37775549 10.1038/s41467-023-41710-y PMC10541888

[51] Tao, L. C. et al. Microglia modulation with 1070-nm light attenuates Aβ burden and cognitive impairment in Alzheimer’s disease mouse model. *Light Sci. Appl.* **10**, 179 (2021). 34493703 10.1038/s41377-021-00617-3 PMC8423759

[52] Kathe, C. et al. Wireless closed-loop optogenetics across the entire dorsoventral spinal cord in mice. *Nat. Biotechnol.* **40**, 198–208 (2022). 34580478 10.1038/s41587-021-01019-x PMC7612390

[53] Zhang, Y. et al. Battery-free, fully implantable optofluidic cuff system for wireless optogenetic and pharmacological neuromodulation of peripheral nerves. *Sci. Adv.* **5**, eaaw5296 (2019). 31281895 10.1126/sciadv.aaw5296 PMC6611690

[54] Liu, X. Y. et al. Fatigue-resistant hydrogel optical fibers enable peripheral nerve optogenetics during locomotion. *Nat. Methods* **20**, 1802–1809 (2023). 37857906 10.1038/s41592-023-02020-9 PMC11009937

[55] Kim, H. et al. Benefits of a skull-interfaced flexible and implantable multilight emitting diode array for photobiomodulation in ischemic stroke. *Adv. Sci.* **9**, e2104629 (2022). 10.1002/advs.202104629 PMC9008794 35076161

[56] Hee Lee, J. et al. Implantable micro-light-emitting diode (µLED)-based optogenetic interfaces toward human applications. *Adv. Drug Deliv. Rev.* **187**, 114399 (2022). 35716898 10.1016/j.addr.2022.114399

[57] Ouyang, W. et al. A wireless and battery-less implant for multimodal closed-loop neuromodulation in small animals. *Nat. Biomed. Eng.* **7**, 1252–1269 (2023). 37106153 10.1038/s41551-023-01029-x

[58] Yamagishi, K. et al. Tissue-adhesive wirelessly powered optoelectronic device for metronomic photodynamic cancer therapy. *Nat. Biomed. Eng.* **3**, 27–36 (2019). 30932063 10.1038/s41551-018-0261-7

[59] Chai, R. Z. & Zhang, Y. Adaptive thermal management of implantable device. *IEEE Sens. J.* **19**, 1176–1185 (2019).

[60] Zhang, Z. et al. High-brightness all-polymer stretchable LED with charge-trapping dilution. *Nature* **603**, 624–630 (2022). 35322250 10.1038/s41586-022-04400-1

[61] Bian, Y. Y. et al. Efficient green InP-based QD-LED by controlling electron injection and leakage. *Nature* **635**, 854–859 (2024). 39567695 10.1038/s41586-024-08197-z

[62] Lee, J. H. et al. Wearable surface-lighting micro-light-emitting diode patch for melanogenesis inhibition. *Adv. Healthc. Mater.* **12**, 2201796 (2023). 10.1002/adhm.202201796 36189834

[63] Lee, J. et al. Stretchable and suturable fibre sensors for wireless monitoring of connective tissue strain. *Nat. Electron.* **4**, 291–301 (2021).

[64] Negri, L. B. et al. An antimicrobial blue light prototype device controls infected wounds in a preclinical porcine model. *J. Infect. Dis.* **231**, e545–e552 (2024). 10.1093/infdis/jiae548 PMC11911787 39535214

[65] Jung, Y. H. et al. A wireless haptic interface for programmable patterns of touch across large areas of the skin. *Nat. Electron.* **5**, 374–385 (2022).

[66] Tang, X. et al. Flexible brain-computer interfaces. *Nat. Electron.* **6**, 109–118 (2023).

[67] Cai, X. et al. A wireless optoelectronic probe to monitor oxygenation in deep brain tissue. *Nat. Photonics* **18**, 492–500 (2024).

[68] Lee, G. H. et al. Multifunctional materials for implantable and wearable photonic healthcare devices. *Nat. Rev. Mater.* **5**, 149–165 (2020). 32728478 10.1038/s41578-019-0167-3 PMC7388681

[69] Mariello, M. et al. Recent advances in encapsulation of flexible bioelectronic implants: materials, technologies, and characterization methods. *Adv. Mater.* **34**, 2201129 (2022). 10.1002/adma.202201129 35353928

[70] He, J. Q. et al. Scalable production of high-performing woven lithium-ion fibre batteries. *Nature* **597**, 57–63 (2021). 34471277 10.1038/s41586-021-03772-0

[71] Zhu, G. Z. et al. Rechargeable Na/Cl_2_ and Li/Cl_2_ batteries. *Nature* **596**, 525–530 (2021). 34433941 10.1038/s41586-021-03757-z

[72] Davies, N. & Wilson, B. C. Interstitial in vivo ALA-PpIX mediated metronomic photodynamic therapy (mPDT) using the CNS-1 astrocytoma with bioluminescence monitoring. *Photodiagn. Photodyn. Ther.* **4**, 202–212 (2007). 10.1016/j.pdpdt.2007.06.002 25047439

[73] Davies, N. & Wilson, B. C. Tetherless fiber-coupled optical sources for extended metronomic photodynamic therapy. *Photodiagn. Photodyn. Ther.* **4**, 184–189 (2007). 10.1016/j.pdpdt.2007.03.005 25047436

[74] Tan, C. X. et al. A high performance wearable strain sensor with advanced thermal management for motion monitoring. *Nat. Commun.* **11**, 3530 (2020). 32669576 10.1038/s41467-020-17301-6 PMC7363829

[75] Tao, P. D. et al. Enhancement of in-plane thermal conductivity of flexible boron nitride heat spreaders by micro/nanovoid filling using deformable liquid metal nanoparticles. *Rare Met.* **42**, 3662–3672 (2023).

[76] Xu, S. D. et al. Realizing a 10 °C cooling effect in a flexible thermoelectric cooler using a vortex generator. *Adv. Mater.* **34**, 2204508 (2022). 10.1002/adma.202204508 36016514

[77] van Erp, R. et al. Co-designing electronics with microfluidics for more sustainable cooling. *Nature* **585**, 211–216 (2020). 32908265 10.1038/s41586-020-2666-1

[78] Min, J. H. et al. An autonomous wearable biosensor powered by a perovskite solar cell. *Nat. Electron.* **6**, 630–641 (2023). 38465017 10.1038/s41928-023-00996-y PMC10923186

[79] Tehrani, F. et al. An integrated wearable microneedle array for the continuous monitoring of multiple biomarkers in interstitial fluid. *Nat. Biomed. Eng.* **6**, 1214–1224 (2022). 35534575 10.1038/s41551-022-00887-1

[80] Xu, Y. C. et al. In-ear integrated sensor array for the continuous monitoring of brain activity and of lactate in sweat. *Nat. Biomed. Eng.* **7**, 1307–1320 (2023). 37770754 10.1038/s41551-023-01095-1 PMC10589098

[81] Zhang, S. P. et al. On-skin ultrathin and stretchable multifunctional sensor for smart healthcare wearables. *npj Flex. Electron.* **6**, 11 (2022).

[82] Zhou, J. et al. Multiscale and hierarchical wrinkle enhanced graphene/Ecoflex sensors integrated with human-machine interfaces and cloud-platform. *npj Flex. Electron.* **6**, 55 (2022). 37520266 10.1038/s41528-022-00189-1 PMC9255543

[83] Zhang, L. D. et al. Advanced and readily-available wireless-powered blue-light-implant for non-invasive peri-implant disinfection. *Adv. Sci.* **10**, 2203472 (2023). 10.1002/advs.202203472 PMC10190665 36935373

[84] Bo, R. H. et al. Mechanically-guided 3D assembly for architected flexible electronics. *Chem. Rev.* **123**, 11137–11189 (2023). 37676059 10.1021/acs.chemrev.3c00335 PMC10540141

[85] Kong, M. et al. Transparent omni-directional stretchable circuit lines made by a junction-free grid of expandable Au lines. *Adv. Mater.* **33**, 2100299 (2021). 10.1002/adma.202100299 34155682

[86] Shen, Q. C. et al. Liquid metal-based soft, hermetic, and wireless-communicable seals for stretchable systems. *Science* **379**, 488–493 (2023). 36730410 10.1126/science.ade7341

[87] Lee, W. et al. Universal assembly of liquid metal particles in polymers enables elastic printed circuit board. *Science* **378**, 637–641 (2022). 36356149 10.1126/science.abo6631

[88] Liu, S. L. Z., Shah, D. S. & Kramer-Bottiglio, R. Highly stretchable multilayer electronic circuits using biphasic gallium-indium. *Nat. Mater.* **20**, 851–858 (2021). 33603186 10.1038/s41563-021-00921-8

[89] Zheng, Y. et al. Environmentally stable and stretchable polymer electronics enabled by surface-tethered nanostructured molecular-level protection. *Nat. Nanotechnol.* **18**, 1175–1184 (2023). 37322142 10.1038/s41565-023-01418-y

[90] Min, H. et al. Additive treatment yields high-performance lead-free perovskite light-emitting diodes. *Nat. Photonics* **17**, 755–760 (2023).

[91] Su, R. T. et al. 3D-printed flexible organic light-emitting diode displays. *Sci. Adv.* **8**, eabl8798 (2022). 34995118 10.1126/sciadv.abl8798 PMC8741182

[92] Chen, C. S. et al. Perovskite solar cells based on screen-printed thin films. *Nature* **612**, 266–271 (2022). 36352221 10.1038/s41586-022-05346-0

[93] Wang, S. C. et al. Inkjet-printed xerogel scaffolds enabled room-temperature fabrication of high-quality metal electrodes for flexible electronics. *Adv. Funct. Mater.* **32**, 2203730 (2022).

[94] Liu, G. Q. et al. Evolution of dip-pen nanolithography (DPN): from molecular patterning to materials discovery. *Chem. Rev.* **120**, 6009–6047 (2020). 32319753 10.1021/acs.chemrev.9b00725

[95] Lee, J. H. et al. Deeply implantable, shape-morphing, 3D MicroLEDs for pancreatic cancer therapy. *Adv. Mater*. 10.1002/adma.202411494 (2024). 10.1002/adma.202411494 39679727

[96] Lin, Q. H. et al. Flexible quantum dot light-emitting device for emerging multifunctional and smart applications. *Adv. Mater.* **35**, 2210385 (2023). 10.1002/adma.202210385 36880739

[97] Jang, E. & Jang, H. Review: quantum dot light-emitting diodes. *Chem. Rev.* **123**, 4663–4692 (2023). 36795794 10.1021/acs.chemrev.2c00695

[98] Zhao, H. N. et al. Stable blue phosphorescent organic LEDs that use polariton-enhanced Purcell effects. *Nature* **626**, 300–305 (2024). 38122821 10.1038/s41586-023-06976-8

[99] Cho, H. H. et al. Suppression of Dexter transfer by covalent encapsulation for efficient matrix-free narrowband deep blue hyperfluorescent OLEDs. *Nat. Mater.* **23**, 519–526 (2024). 38480865 10.1038/s41563-024-01812-4 PMC10990937

[100] Kim, T. et al. Efficient and stable blue quantum dot light-emitting diode. *Nature* **586**, 385–389 (2020). 33057219 10.1038/s41586-020-2791-x

[101] Ryu, J. E. et al. Technological breakthroughs in chip fabrication, transfer, and color conversion for high-performance micro-LED displays. *Adv. Mater.* **35**, 2204947 (2023). 10.1002/adma.202204947 35950613

[102] Chang, W. et al. Concurrent self-assembly of RGB microLEDs for next-generation displays. *Nature* **617**, 287–291 (2023). 37138079 10.1038/s41586-023-05889-w

[103] Huang, J. S. et al. Near-infrared photodynamic chemiluminescent probes for cancer therapy and metastasis detection. *Angew. Chem. Int. Ed.* **62**, e202303982 (2023). 10.1002/anie.202303982 37050864

[104] Keum, C. et al. A substrateless, flexible, and water-resistant organic light-emitting diode. *Nat. Commun.* **11**, 6250 (2020). 33288769 10.1038/s41467-020-20016-3 PMC7721873

[105] Kim, J. H. & Park, J. W. Intrinsically stretchable organic light-emitting diodes. *Sci. Adv.* **7**, eabd9715 (2021). 33627424 10.1126/sciadv.abd9715 PMC7904263

[106] Huang, T. Y. et al. Delocalizing electron distribution in thermally activated delayed fluorophors for high-efficiency and long-lifetime blue electroluminescence. *Nat. Mater.* **23**, 1523–1530 (2024). 39266678 10.1038/s41563-024-02004-w

[107] Hua, T. et al. Deep-blue organic light-emitting diodes for ultrahigh-definition displays. *Nat. Photonics* **18**, 1161–1169 (2024).

[108] Zhao, H. N., Arneson, C. E. & Forrest, S. R. Stable, deep blue tandem phosphorescent organic light-emitting diode enabled by the double-sided polariton-enhanced Purcell effect. *Nat. Photonics* **19**, 607–614 (2025).

[109] Tankelevičiūtė, E., Samuel, I. D. W. & Zysman-Colman, E. The blue problem: OLED stability and degradation mechanisms. *J. Phys. Chem. Lett.* **15**, 1034–1047 (2024). 38259039 10.1021/acs.jpclett.3c03317 PMC10839906

[110] Choi, D. K. et al. Highly efficient, heat dissipating, stretchable organic light-emitting diodes based on a MoO_3_/Au/MoO_3_ electrode with encapsulation. *Nat. Commun.* **12**, 2864 (2021). 34001906 10.1038/s41467-021-23203-y PMC8128878

[111] Cha, G. D., Kim, D. H. & Kim, D. C. Wearable and implantable light-emitting diodes and their biomedical applications. *Korean J. Chem. Eng.* **41**, 1–24 (2024).

[112] Chen, H. et al. Flexible quantum dot light-emitting devices for targeted photomedical applications. *J. Soc. Inf. Disp.* **26**, 296–303 (2018). 30416331 10.1002/jsid.650 PMC6223313

[113] Chen, H. et al. Quantum dot light emitting devices for photomedical applications. *J. Soc. Inf. Disp.* **25**, 177–184 (2017). 28867926 10.1002/jsid.543 PMC5576728

[114] Kim, Y. W. et al. Wearable quantum dots organic light-emitting diodes patch for high-power near infra-red photomedicene with real-time wavelength control. *Chem. Eng. J.* **499**, 156121 (2024).

[115] Tri, T. T. et al. Maximization of cytochrome C oxidase enzyme activity by optimizing color conversion from red organic light-emitting diodes. *Appl. Mater. Today* **38**, 102223 (2024).

[116] Wang, Y. K. et al. Long-range order enabled stability in quantum dot light-emitting diodes. *Nature* **629**, 586–591 (2024). 38720080 10.1038/s41586-024-07363-7

[117] Kang, K. S. et al. Reliable high temperature, high humidity flexible thin film encapsulation using Al_2_O_3_/MgO nanolaminates for flexible OLEDs. *Nano Res.* **13**, 2716–2725 (2020).

[118] Kishore, R. A. et al. Ultra-high performance wearable thermoelectric coolers with less materials. *Nat. Commun.* **10**, 1765 (2019). 30992438 10.1038/s41467-019-09707-8 PMC6468009

[119] Liu, H. et al. Development and evaluation of a low-cost, portable, LED-based device for PDT treatment of early-stage oral cancer in resource-limited settings. *Lasers Surg. Med.* **51**, 345–351 (2019). 30168618 10.1002/lsm.23019 PMC6934354

[120] Lu, C. H. et al. High-performance fibre battery with polymer gel electrolyte. *Nature* **629**, 86–91 (2024). 38658763 10.1038/s41586-024-07343-x

[121] Zhang, Y. J. et al. A microscale soft lithium-ion battery for tissue stimulation. *Nat. Chem. Eng.* **1**, 691–701 (2024). 39620147 10.1038/s44286-024-00136-z PMC11606923

[122] Hong, Y. et al. Energetic and durable all-polymer aqueous battery for sustainable, flexible power. *Nat. Commun.* **15**, 9539 (2024). 39496602 10.1038/s41467-024-53804-2 PMC11535528

[123] Zhang, C. et al. Conjunction of triboelectric nanogenerator with induction coils as wireless power sources and self-powered wireless sensors. *Nat. Commun.* **11**, 58 (2020). 31896757 10.1038/s41467-019-13653-w PMC6940365

[124] Seo, D. et al. Wireless recording in the peripheral nervous system with ultrasonic neural dust. *Neuron* **91**, 529–539 (2016). 27497221 10.1016/j.neuron.2016.06.034

[125] Ding, H. et al. Microscale optoelectronic infrared-to-visible upconversion devices and their use as injectable light sources. *Proc. Natl Acad. Sci. USA* **115**, 6632–6637 (2018). 29891705 10.1073/pnas.1802064115 PMC6042105

[126] Park, S. I. et al. Soft, stretchable, fully implantable miniaturized optoelectronic systems for wireless optogenetics. *Nat. Biotechnol.* **33**, 1280–1286 (2015). 26551059 10.1038/nbt.3415 PMC4880021

[127] Liu, Z. et al. Human motion driven self-powered photodynamic system for long-term autonomous cancer therapy. *ACS Nano* **14**, 8074–8083 (2020). 32551540 10.1021/acsnano.0c00675

[128] Jeong, Y. C. et al. Progress in brain-compatible interfaces with soft nanomaterials. *Adv. Mater.* **32**, 1907522 (2020). 10.1002/adma.201907522 32297395

[129] Kim, T. I. et al. Injectable, cellular-scale optoelectronics with applications for wireless optogenetics. *Science* **340**, 211–216 (2013). 23580530 10.1126/science.1232437 PMC3769938

[130] Yang, X. et al. Bioinspired neuron-like electronics. *Nat. Mater.* **18**, 510–517 (2019). 30804509 10.1038/s41563-019-0292-9 PMC6474791

[131] Zhang, Z. T. Light-emitting materials for wearable electronics. *Nat. Rev. Mater.* **7**, 839–840 (2022).

[132] Ma, D. X. et al. Distribution control enables efficient reduced-dimensional perovskite LEDs. *Nature* **599**, 594–598 (2021). 34819678 10.1038/s41586-021-03997-z

[133] Shin, J. et al. Vertical full-colour micro-LEDs via 2D materials-based layer transfer. *Nature* **614**, 81–87 (2023). 36725999 10.1038/s41586-022-05612-1

[134] Kim, D. C. et al. Intrinsically stretchable quantum dot light-emitting diodes. *Nat. Electron.* **7**, 365–374 (2024).

[135] Si, M. W. et al. Scaled indium oxide transistors fabricated using atomic layer deposition. *Nat. Electron.* **5**, 164–170 (2022).

[136] Chorsi, M. T. et al. Highly piezoelectric, biodegradable, and flexible amino acid nanofibers for medical applications. *Sci. Adv.* **9**, eadg6075 (2023). 37315129 10.1126/sciadv.adg6075 PMC10266740

[137] Zhang, Y. P. et al. Flexible self-powered integrated sensing system with 3D periodic ordered black phosphorus@MXene thin-films. *Adv. Mater.* **33**, 2007890 (2021). 10.1002/adma.202007890 33899274

[138] Sani, E. S. et al. A stretchable wireless wearable bioelectronic system for multiplexed monitoring and combination treatment of infected chronic wounds. *Sci. Adv.* **9**, eadf7388 (2023). 36961905 10.1126/sciadv.adf7388 PMC10038347

[139] Bidinger, S. L. et al. Pulsed transistor operation enables miniaturization of electrochemical aptamer-based sensors. *Sci. Adv.* **8**, eadd4111 (2022). 36383656 10.1126/sciadv.add4111 PMC9668304

[140] Wu, H. et al. On-skin biosensors for noninvasive monitoring of postoperative free flaps and replanted digits. *Sci. Transl. Med.* **15**, eabq1634 (2023). 37099631 10.1126/scitranslmed.abq1634

[141] Lee, J. H. et al. 3D printed, customizable, and multifunctional smart electronic eyeglasses for wearable healthcare systems and human-machine interfaces. *Acs Appl. Mater. Interfaces* **12**, 21424–21432 (2020). 32319751 10.1021/acsami.0c03110

[142] Kim, S. K. et al. Bimetallic nanocatalysts immobilized in nanoporous hydrogels for long-term robust continuous glucose monitoring of smart contact lens. *Adv. Mater.* **34**, 2110536 (2022). 10.1002/adma.202110536 PMC10782562 35194844

[143] Kim, J. et al. A soft and transparent contact lens for the wireless quantitative monitoring of intraocular pressure. *Nat. Biomed. Eng.* **5**, 772–782 (2021). 33941897 10.1038/s41551-021-00719-8

[144] Liu, W. J. et al. Neuroprosthetic contact lens enabled sensorimotor system for point-of-care monitoring and feedback of intraocular pressure. *Nat. Commun.* **15**, 5635 (2024). 38965218 10.1038/s41467-024-49907-5 PMC11224243

[145] Cardoso, F. D. S., Gonzalez-Lima, F. & Gomes da Silva, S. Photobiomodulation for the aging brain. *Ageing Res. Rev.* **70**, 101415 (2021). 34325071 10.1016/j.arr.2021.101415

[146] Cunnane, S. C. et al. Brain energy rescue: an emerging therapeutic concept for neurodegenerative disorders of ageing. *Nat. Rev. Drug Discov.* **19**, 609–633 (2020). 32709961 10.1038/s41573-020-0072-x PMC7948516

[147] Aini, N. et al. The effects of light therapy on sleep, depression, neuropsychiatric behaviors, and cognition among people living with dementia: a meta-analysis of randomized controlled trials. *Am. J. Geriatr. Psychiatry* **32**, 681–706 (2024). 38216355 10.1016/j.jagp.2023.12.010

[148] Yin, L. et al. A stretchable epidermal sweat sensing platform with an integrated printed battery and electrochromic display. *Nat. Electron.* **5**, 694–705 (2022).

[149] Wainwright, M. et al. Photoantimicrobials-are we afraid of the light? *Lancet Infect. Dis.* **17**, E49–E55 (2017). 27884621 10.1016/S1473-3099(16)30268-7 PMC5280084

[150] Ju, X. K. et al. A wearable electrostimulation-augmented ionic-gel photothermal patch doped with MXene for skin tumor treatment. *Nat. Commun.* **15**, 762 (2024). 38278810 10.1038/s41467-024-45070-z PMC10817919

[151] Sempionatto, J. R. et al. An epidermal patch for the simultaneous monitoring of haemodynamic and metabolic biomarkers. *Nat. Biomed. Eng.* **5**, 737–748 (2021). 33589782 10.1038/s41551-021-00685-1

[152] Schalkamp, A. K. et al. Wearable movement-tracking data identify Parkinson’s disease years before clinical diagnosis. *Nat. Med.* **29**, 2048–2056 (2023). 37400639 10.1038/s41591-023-02440-2

[153] Chung, H. U. et al. Skin-interfaced biosensors for advanced wireless physiological monitoring in neonatal and pediatric intensive-care units. *Nat. Med.* **26**, 418–429 (2020). 32161411 10.1038/s41591-020-0792-9 PMC7315772

[154] Chung, H. U. et al. Binodal, wireless epidermal electronic systems with in-sensor analytics for neonatal intensive care. *Science* **363**, eaau0780 (2019). 30819934 10.1126/science.aau0780 PMC6510306

[155] Inamori, G. et al. Neonatal wearable device for colorimetry-based real-time detection of jaundice with simultaneous sensing of vitals. *Sci. Adv.* **7**, eabe3793 (2021). 33658197 10.1126/sciadv.abe3793 PMC7929506

[156] Hillebrandt, S. et al. High brightness, highly directional organic light-emitting diodes as light sources for future light-amplifying prosthetics in the optogenetic management of vision loss. *Adv. Opt. Mater.* **11**, 2200877 (2022).

[157] Lee, G. H. et al. Smart wireless near-infrared light emitting contact lens for the treatment of diabetic retinopathy. *Adv. Sci.* **9**, 2106254 (2022). 10.1002/advs.202103254 PMC8948592 35092362

[158] Sun, S. Q. et al. Red organic light-emitting diodes based photobiomodulation therapy enabling prominent hair growth. *Nano Res.* **16**, 7164–7170 (2023).

[159] Jeon, Y. et al. Parallel-stacked flexible organic light-emitting diodes for wearable photodynamic therapeutics and color-tunable optoelectronics. *ACS Nano* **14**, 15688–15699 (2020). 33155466 10.1021/acsnano.0c06649

[160] Lian, C. et al. Flexible organic light-emitting diodes for antimicrobial photodynamic therapy. *npj Flex. Electron.* **3**, 18 (2019).

[161] Song, J. et al. Organic light-emitting diodes: pushing toward the limits and beyond. *Adv. Mater.* **32**, 1907539 (2020). 10.1002/adma.201907539 32142190

[162] Kang, S. K. et al. Bioresorbable silicon electronic sensors for the brain. *Nature* **530**, 71–76 (2016). 26779949 10.1038/nature16492

[163] Wang, C. H. et al. Monitoring of the central blood pressure waveform via a conformal ultrasonic device. *Nat. Biomed. Eng.* **2**, 687–695 (2018). 30906648 10.1038/s41551-018-0287-x PMC6428206

[164] Xu, C. H. et al. A physicochemical-sensing electronic skin for stress response monitoring. *Nat. Electron.* **7**, 168–179 (2024). 38433871 10.1038/s41928-023-01116-6 PMC10906959

[165] Juengpanich, S. et al. Pre-activated nanoparticles with persistent luminescence for deep tumor photodynamic therapy in gallbladder cancer. *Nat. Commun.* **14**, 5699 (2023). 37709778 10.1038/s41467-023-41389-1 PMC10502062

[166] Tang, Y. F. et al. Oxygen-independent organic photosensitizer with ultralow-power NIR photoexcitation for tumor-specific photodynamic therapy. *Nat. Commun.* **15**, 2530 (2024). 38514624 10.1038/s41467-024-46768-w PMC10957938

[167] Chen, J. et al. Atomically precise photothermal nanomachines. *Nat. Mater.* **23**, 271–280 (2024). 37957270 10.1038/s41563-023-01721-y

[168] Zhang, Q. et al. Noninvasive low-level laser therapy for thrombocytopenia. *Sci. Transl. Med.* **8**, 349ra101 (2016). 10.1126/scitranslmed.aaf4964 PMC7392149 27464749

[169] Wang, Z. H. et al. Adoptive macrophage directed photodynamic therapy of multidrug-resistant bacterial infection. *Nat. Commun.* **14**, 7251 (2023). 37945555 10.1038/s41467-023-43074-9 PMC10636156

[170] Xiu, W. et al. Potentiating hypoxic microenvironment for antibiotic activation by photodynamic therapy to combat bacterial biofilm infections. *Nat. Commun.* **13**, 3875 (2022). 35790729 10.1038/s41467-022-31479-x PMC9256606

[171] Li, G. Q. et al. Three-dimensional flexible electronics using solidified liquid metal with regulated plasticity. *Nat. Electron.* **6**, 154–163 (2023).

[172] Verboven, I. & Deferme, W. Printing of flexible light emitting devices: a review on different technologies and devices, printing technologies and state-of-the-art applications and future prospects. *Prog. Mater. Sci.* **118**, 100760 (2021).

[173] Koo, J. H. et al. A vacuum-deposited polymer dielectric for wafer-scale stretchable electronics. *Nat. Electron.* **6**, 137–145 (2023).

[174] Won, S. M. et al. Wireless and battery-free technologies for neuroengineering. *Nat. Biomed. Eng.* **7**, 405–423 (2023). 33686282 10.1038/s41551-021-00683-3 PMC8423863

[175] Yang, S. L. X. et al. GSH/pH dual activatable cross-linked and fluorinated PEI for cancer gene therapy through endogenous iron De-hijacking and in situ ROS amplification. *Adv. Mater.* **36**, 2304098 (2024). 10.1002/adma.202304098 37689975

